# Penidihydrocitrinins A–C: New Polyketides from the Deep-Sea-Derived *Penicillium citrinum* W17 and Their Anti-Inflammatory and Anti-Osteoporotic Bioactivities

**DOI:** 10.3390/md21100538

**Published:** 2023-10-14

**Authors:** Yong Zhang, Chun-Lan Xie, Yuan Wang, Xi-Wen He, Ming-Min Xie, You Li, Kai Zhang, Zheng-Biao Zou, Long-He Yang, Ren Xu, Xian-Wen Yang

**Affiliations:** 1 Key Laboratory of Marine Genetic Resources, Technical Innovation Center for Utilization of Marine Biological Resources, Third Institute of Oceanography, Ministry of Natural Resources, 184 Daxue Road, Xiamen 361005, China; zhangyong@tio.org.cn (Y.Z.); xiechunlanxx@163.com (C.-L.X.); wy2012016284@163.com (Y.W.); hexiwen1224@163.com (X.-W.H.); xiemingmin@tio.org.cn (M.-M.X.); m220200718@st.shou.edu.cn (Y.L.); z18252730063@163.com (K.Z.); zhengbiaozou@njust.edu.cn (Z.-B.Z.); 2 School of Medicine, Xiamen University, South Xiangan Road, Xiamen 361005, China

**Keywords:** deep-sea, fungus, *Penicillium citrinum*, polyketides, anti-osteoporosis, anti-inflammation

## Abstract

Three new polyketides (penidihydrocitrinins A–C, **1**–**3**) and fourteen known compounds (**4**–**17**) were isolated from the deep-sea-derived *Penicillium citrinum* W17. Their structures were elucidated by comprehensive analyses of 1D and 2D NMR, HRESIMS, and ECD calculations. Compounds **1**–**17** were evaluated for their anti-inflammatory and anti-osteoporotic bioactivities. All isolates exhibited significant inhibitory effects on LPS-stimulated nitric oxide production in murine brain microglial BV-2 cells in a dose-response manner. Notably, compound **14** displayed the strongest effect with the IC_50_ value of 4.7 µM. Additionally, compounds **6**, **7**, and **8** significantly enhanced osteoblast mineralization, which was comparable to that of the positive control, purmorphamine. Furthermore, these three compounds also suppressed osteoclastogenesis in a dose-dependent manner under the concentrations of 2.5 μM, 5.0 μM, and 10 μM.

## 1. Introduction

Marine microbes are an ideal source to yield diverse secondary metabolites with unprecedented structures. As a matter of fact, about half of the new marine natural products are produced by marine microorganisms [1,2,3], especially those living in the deep sea under extremely tough environments such as low oxygen concentration, high salt, high hydrostatic pressure, and absence of light, which require various biochemical and physiological adaptations for survival [4,5]. These adaptions are accompanied by adjustments of gene regulation, resulting in the formation of different metabolic pathways to give birth to a large number of new secondary metabolites [4,5].

Polyketides are a class of structurally diverse natural products with carbon skeletons originating from the polymerization of short-chain carboxylic acids units, including acetate, propionate, butyrate, etc. [6], which are catalyzed by polyketide synthases (PKSs) [7]. Polyketides have attracted wide attention due to their promising bioactivities [8,9]. For example, salinosporamide A showed significant proteasomal chymotrypsin-like proteolytic inhibitory activity with an IC_50_ value of 1.3 nM [10]; microketide A exhibited remarkable antibacterial activities against *Pseudomonas aeruginosa*, *Nocardia brasiliensis*, *Kocuria rhizophila*, and *Bacillus anthraci* with an equal minimum inhibitory concentration (MIC) value (0.19 μg/mL) to that of ciprofloxacin [11]; and theissenone exhibited potent nitric oxide production inhibitory activity in murine brain microglial BV-2 cells with an IC_50_ value of 5.0 ± 1.0 μM [12]. As some of the most abundant fungi of the world, *Penicillium* species could generate a broad spectrum of unique polyketides. For instance, two new tricyclic polyketides, penijanthinones A and B, were isolated from *P*. *janthinellum* HK1-6 [13,14]; two new C-8 benzoyl-substituted azaphilones, pinazaphilones A and B, were obtained from *Penicillium* sp. HN29-3B1 [13,14]; chloctanspirones A and B, possessing an unprecedented bicyclo [2.2.2] octane-2-spiro cyclohexane skeleton, were discovered in *P*. *terrestre* [15].

**Figure 1:**
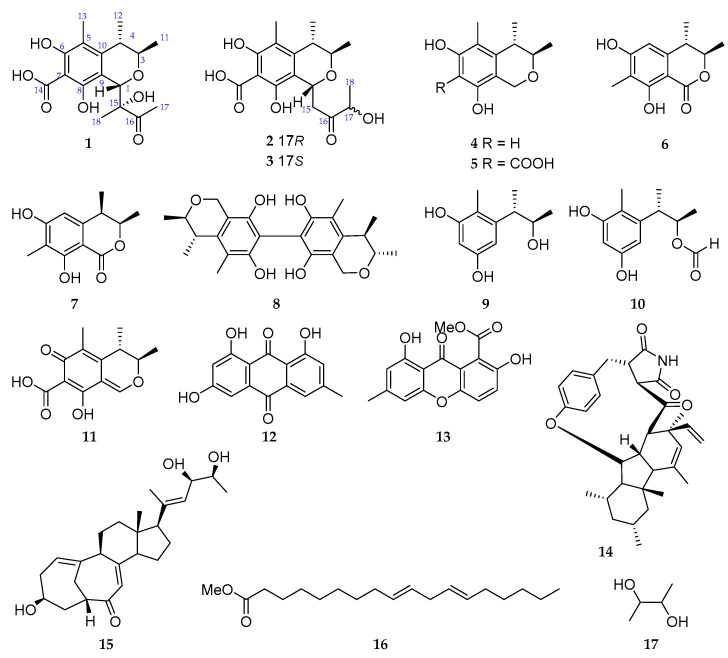
Compounds **1**–**17** isolated from the deep-sea-derived *Penicillium citrinum* W17.

As part of our continuing discovery of structurally novel and biologically interesting compounds from deep-sea-derived microorganisms [16,17,18,19,20], the crude extract of *Penicillium citrinum* W17 isolated from a deep-sea sediment sample (−5278 m) of the western Pacific Ocean revealed the rich chemical diversity of the secondary metabolites. Therefore, it was subjected to a systematic chemical investigation. As a result, three new polyketides (penidihydrocitrinins A–C, **1**–**3**) and fourteen known compounds (**4**–**17**) were obtained (Figure 1). Herein, we report the details of isolation, structure elucidation, and bioactivities of these isolates. 

## 2. Results and Discussion

The EtOAc-soluble extract of the deep-sea-derived *Penicillium citrinum* W17 was subjected to column chromatography (CC) on silica gel, ODS, and Sephadex LH-20, as well as preparative HPLC to afford 17 compounds (**1**–**17**).

**Figure 2:**
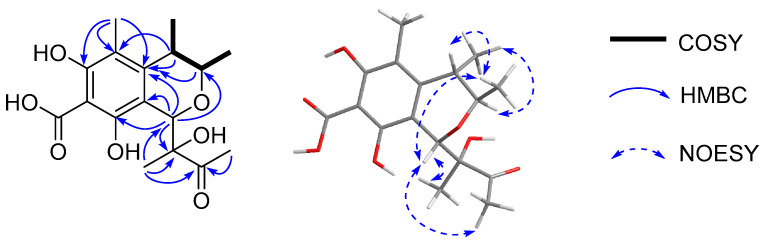
The key COSY, HMBC, and NOESY correlations of compound **1**.

Compound **1** was obtained as yellow amorphous powder. The molecular formula was determined as C_17_H_22_O_7_ with seven degrees of unsaturation (DoU) on the basis of HRESIMS spectrum at *m*/*z* 337.1309 [M − H]^−^ (Figure S1 of the Supplementary Materials). The ^1^H NMR spectroscopic data (Table 1) showed two methyl doublets (*δ*_H_ 1.11 (d, *J* = 6.4 Hz, 11-Me) and 1.28 (d, *J* = 6.9 Hz, 12-Me)), three methyl singlets (*δ*_H_ 1.10 (s, 18-Me), 2.04 (s, 13-Me), and 2.30 (s, 17-Me)), and three methines (*δ*_H_ 2.62 (qd, *J* = 6.7 Hz, 1.8 Hz, H-4), 3.98 (qd, *J* = 6.9 Hz, 1.8 Hz, H-3), 5.17 (s, H-1)). The ^13^C NMR spectroscopic data exhibited 17 carbon resonance signals, including five methyls (*δ*_C_ 10.2 (q, 13-Me), 18.3 (q, 11-Me), 20.1 (q, 12-Me), 20.7 (q, 18-Me), 25.0 (q, 17-Me)), three methines ((*δ*_C_ 36.9 (d, C-4), 74.0 (d, C-3), 75.4 (d, C-1)), and nine nonprotonated carbons (*δ*_C_ 83.2 (s, C-15), 102.2 (s, C-7), 110.3 (s, C-9), 114.0 (s, C-5), 143.8 (s, C-10), 156.7 (s, C-8), 160.0 (s, C-6), 178.2 (s, C-14), 211.3 (s, C-16)). The COSY correlations of 11-Me/H-3/H-4/12-Me, together with the HMBC correlations from H-4 to C-5 and C-10, from 13-Me to C-5, C-6, and C-10, from H-3 to C-10, and from H-1 to C-3, C-8, C-9, and C-10, constructed a dihydrocitrinin fragment. In combination with the HMBC cross peaks of 17-Me with C-16 and of 18-Me with C-1, C-15, and C-16, the planar structure of compound **1** was established as 2-hydroxy-3-butonyldihydrocitrinin (Figure 2).

**Figure 3:**
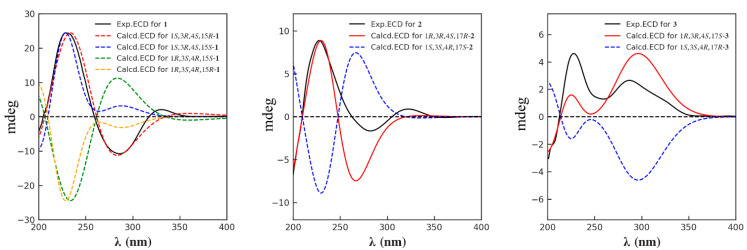
The calculated and experimental ECD spectra of compounds **1**–**3**.

The correlations of H-3/12-Me and 11-Me/H-1/H-4 were observed in the NOESY spectrum, indicating H-1, H-4, and 11-Me were on the same plane, opposite to H-3 and 12-Me (Figure 2). Although the NOESY correlations were found of H-1 to 17-Me and 18-Me, revealing the relative configuration of C-15, more solid evidence is needed to confirm the absolute configuration because of the flexible structure of the segment. Accordingly, the theoretical calculations of the electronic circular dichroism (ECD) spectrum of (1*S*,3*R*,4*S*,15*R*)-**1** and (1*S*,3*R*,4*S*,15*S*)-**1** were conducted along with their enantiomers of (1*R*,3*S*,4*R*,15*S*)-**1** and (1*R*,3*S*,4*R*,15*R*)-**1**. As shown in Figure 3, the calculated ECD spectrum of (1*S*,3*R*,4*S*,15*R*)-**1** matched well with the experimental one. On the basis of the above evidence, compound **1** was determined as (1*S*,3*R*,4*S*,15*R*)-2-hydroxy-3-butonyldihydrocitrinin and named penidihydrocitrinin A.

**Figure 4:**
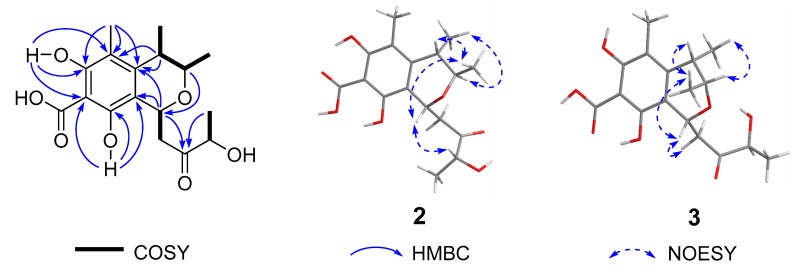
The key COSY, HMBC, and NOESY correlations of compounds **2** and **3**.

Compound **2** was obtained as a colorless oil. Its molecular formula was assigned as C_17_H_22_O_7_ on the basis of HRESIMS data at *m*/*z* 337.1348 [M − H]^−^, suggesting seven degrees of unsaturation. Four methyls (*δ*_H_ 1.01 (d, *J* = 6.5 Hz, 11-Me), 1.17 (d, *J* = 6.8 Hz, 12-Me), 1.21 (d, *J* = 7.0 Hz, 18-Me), and 1.93 (s, 13-Me)) and four methines (*δ*_H_ 2.52 (m, H-4), 3.82 (qd, *J* = 6.6 Hz, 1.5 Hz, H-3), 4.11 (m, H-17), and 5.08 (d, *J* = 9.7 Hz, H-1)) were recognized in the ^1^H NMR spectrum (Figure S9). The ^13^C NMR spectrum in association with the HSQC spectrum indicated 17 carbon signals ascribed to four methyls at *δ*_C_ 9.5 (q, 13-Me), 18.2 (q, 11-Me), 19.2 (q, 18-Me), and 20.1 (q, 12-Me); one methylene at *δ*_C_ 43.1 (t, C-15); four methines at *δ*_C_ 35.1 (d, C-4), 64.8 (d, C-1), 71.7 (d, C-3), and 72.2 (d, C-17); and eight nonprotonated carbons at *δ*_C_ 101.8 (s, C-7), 109.5 (s, C-5), 110.7 (s, C-9), 139.7 (s, C-10), 155.8 (s, C-8), 158.5 (s, C-6), 175.6 (s, C-14), and 211.9 (s, C-16). The spin systems of 11-Me/H-3/H-4/12-Me, H-17/18-Me, and H-1/H-15 were observed in the COSY spectrum, constructing three segments (Figure 4). In the HMBC spectrum, correlations were found of 13-Me to C-5/C-6/C-10, 6-OH to C-5/C-6/C-7, 8-OH to C-7/C-8/C-9, H-4 to C-3/C-5/C-10/C-12, H-1 to C-9/C-10/C-15/C-16, H-3 to C-1, and 18-Me to C-16/C-17. Taking the COSY and HMBC correlations together, the planar structure of compound **2** was then constructed as 3-hydroxy-2-butonyldihydrocitrinin, an isomer of **1**. The relative configuration of **1** was regarded as the same as that of **2** on the basis of the key NOESY correlations of H-1/11-Me, H-3/12-Me, and H-4/11-Me (Figure 4). By the biosynthetic consideration, the absolute configuration of C-1, C-3, and C-4 in **2** and **1** should be the same. However, the stereochemistry of C-17 could not be determined. Therefore, the theoretical calculation of the ECD spectrum was performed. As a result, the experimental ECD spectrum matched well with that of (1*R*,3*R*,4*S*,17*R*)-**2** (Figure 4). Consequently, the structure of **2** was assigned as (1*R*,3*R*,4*S*,17*R*)-3-hydroxy-2-butonyldihydrocitrinin and named penidihydrocitrinin B.

Compound **3** was isolated as a colorless oil. The molecular formula of C_17_H_22_O_7_ was established based on its HRESIMS spectrum at *m*/*z* 337.1327 [M − H]^−^ (calcd for C_17_H_21_O_7_, 337.1287). Its ^1^H and ^13^C NMR spectroscopic data were very similar to those of **2**, except for the upshift of H-17 from *δ*_H_ 4.11 to *δ*_H_ 4.02 and H_3_-18 from *δ*_H_ 1.21 to 1.15 and the downshift of C-17 from *δ*_C_ 72.2 to 72.8. This implied that compound **3** could be an epimer of **2** with *S*-configuration at the C-17 position. The assumption was evidenced by the positive Cotton effect (CE) at *λ*max 287 nm (Δε +0.54) in **3**, whereas a negative CE (Δε −0.34 at λmax 282 nm) in **2**. Final confirmation was obtained by comparison of the calculated and experimental ECD spectra of **3**, showing the calculated ECD spectrum of (1*R*,3*R*,4*S*,17*S*)-**3** was in good accordance with that of the experimental curve (Figure 4). Accordingly, compound **3** was defined as (1*R*,3*R*,4*S*,17*S*)-3-hydroxy-2-butonyldihydrocitrinin, and named penidihydrocitrinin C.

**Scheme 1:**
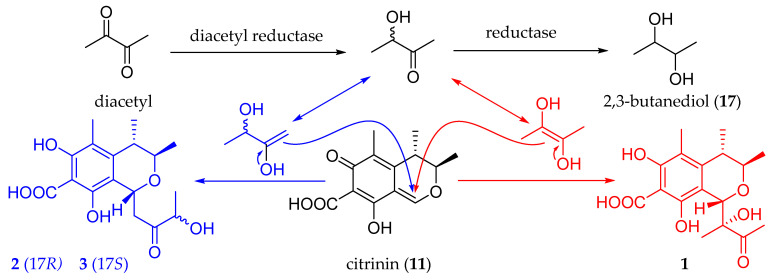
The proposed biosynthetic pathway of penidihydrocitrinins A–C (**1**–**3**).

Compounds **1**–**3** were three novel polyketide adducts of citrinin and diacetyl. They might be biosynthesized by citrinin (**11**) and diacetyl, a widely found secondary metabolite in microorganisms [21] (Scheme 1). Noteworthily, the reduced derivative of diacetyl, 2,3-butanediol (**17**), was also co-isolated from the same extract of the strain.

By comparison of the NMR and MS data with those published in the literature, 14 known compounds were identified as decarboxydihydrocitrinin (**4**) [22], dihydrocitrinin (**5**) [23], (3R*,4S*)-6,8-dihydroxy-3,4,7-trimethylisocoumarin (**6**) [24], sclerotinin C (**7**) [25], asperbiphenyl (**8**) [26], phenol A (**9**) [24], citrinin H2 (**10**) [27], citrinin (**11**) [28], emodin (**12**) [29], pinselin (**13**) [30], GKK1032 B (**14**) [31], neocyclocitrinol C (**15**) [32], (*Z*,*Z*)-9,12-ocadecadienoic acid methyl ester (**16**) [33], and 2,3-butanediol (**17**) [34].

Microglial activation plays a pivotal role in the pathogenesis of neurodegenerative diseases, orchestrating a complex interplay between inflammation and neuronal health [35]. While microglial cells function as the guardians of the central nervous system, their dysregulated activation can lead to chronic neuroinflammation and exacerbation of neuronal damage, contributing to the progression of disorders such as multiple sclerosis and Alzheimer’s and Parkinson’s diseases [36]. These diseases pose significant challenges to public health, which necessitate innovative therapeutic approaches. Thus, discovering new small molecules that can inhibit the dysregulated activation of microglial cells is essential for the targeted modulation of the immune response in the central nervous system, which can reduce inflammation and protect neurons from harm [37]. A growing amount of evidence demonstrates that secondary metabolites derived from marine resources are potential therapeutic strategies for microglial-mediated neuroinflammation.

**Figure 5:**
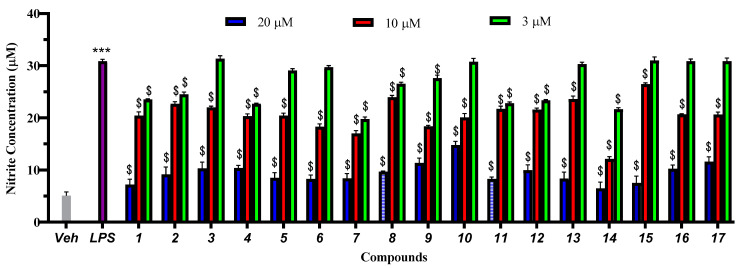
Effects of compounds **1**–**17** against nitrite production in LPS-induced BV-2 cells. *** *p* < 0.0001 vs. Veh; $ *p* < 0.0001 vs. LPS.

**Figure 6:**
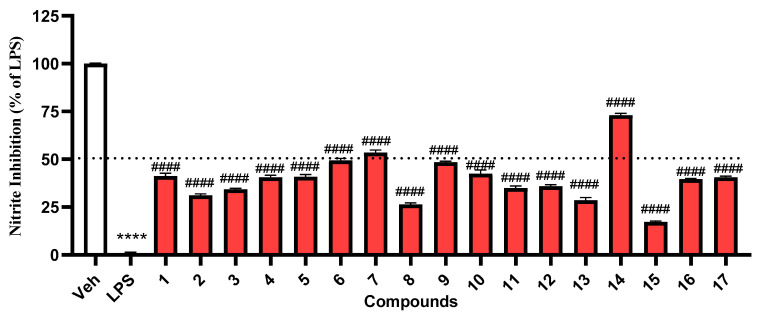
Inhibitory effects of compounds **1**–**17** (10 μM) on LPS-induced nitrite production in BV-2 cells. **** *p* < 0.0001 vs. Veh; #### *p* < 0.0001 vs. LPS.

Therefore, all isolates were tested for nitrite secretion in lipopolysaccharide (LPS)-induced BV-2 microglial cells. As a result, they all demonstrated a dose-dependent suppression of nitrite secretion induced by LPS, displaying inhibitory actions at concentrations of 3.0 µM, 10 µM, and 20 µM (Figure 5). Moreover, none exhibited cytotoxicity effects against BV-2 cells at 20 µM under the microscope. We further compared the inhibition of these compounds on nitrite production at a concentration of 10 μM (Figure 6), and the results showed that the inhibitory rates of compounds **1**–**7** on nitrite production were 31.1–53.5% which indicates that substitution of the C-1 position on isochroman weakened the anti-inflammatory activity of the compounds, suggesting that this substitution is related to anti-inflammatory activity. Compound **8** only inhibited nitrite production by 26.4% in LPS-stimulated BV-2 cells, demonstrating that the dimeric structure further weakened the anti-inflammatory effect. Meanwhile, the anti-inflammatory activity of compound **12**, emodin, was consistent with a previous report [38]. Notably, compound **14** (GKK1032 B) displayed the most potent nitrite inhibitory activity with an inhibitory ratio of 73.0 ± 1.6% at 10 µM (nitrite concentration: 12.2 ± 0.4 µM), compared to the LPS-treated group (nitrite concentration: 30.9 ± 0.4 µM) (Figure 5 and Figure 6). Furthermore, this compound displayed an IC_50_ value of 4.7 µM. Although GKK1032 analogues were reported to exhibit antibacterial activities [39], it is the first time that they had anti-neuroinflammatory activity. These findings demonstrate the effects of marine-derived compounds in modulating microglial activation, suggesting their potential as therapeutic candidates for neuroinflammatory conditions and neurodegenerative diseases.

**Figure 7:**
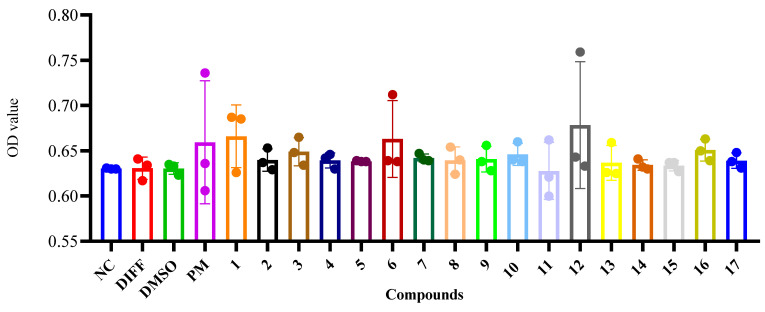
BMSC viability of compounds **1**–**17** was measured by the CCK-8 assay. The culture medium, osteogenesis differentiation medium (50 μg/mL ascorbic acid and 5 mM β-glycerophosphate), DMSO (0.1%), and purmorphamine (1 μM) were regarded as the negative control (NC), differentiation (DIFF), solvent (DMSO), and positive control (PM) groups, respectively.

Osteoporosis, a disease associated with aging, is characterized by excessive activation of osteoclasts or reduction of osteoblasts. Among women aged 65 or older, approximately 25% are affected by osteoporosis, with accelerated bone loss occurring after menopause. Therefore, promoting osteoblast differentiation and suppressing osteoclastogenesis are effective strategies for treating osteoporosis [40]. BMSCs are able to differentiate into osteoblasts, chondroblasts, and adipocytes [41], and bone regeneration achieved via osteogenic induction of MSCs could provide a rational therapeutic strategy for preventing age-related osteoporosis [42]. Accordingly, all 17 isolates were tested for both osteoblast and osteoclast activities. Firstly, compounds **1**–**17** were subjected to anti-proliferative tests on BMSCs. At the concentration of 10 µM, none showed cytotoxicity (Figure 7).

**Figure 8:**
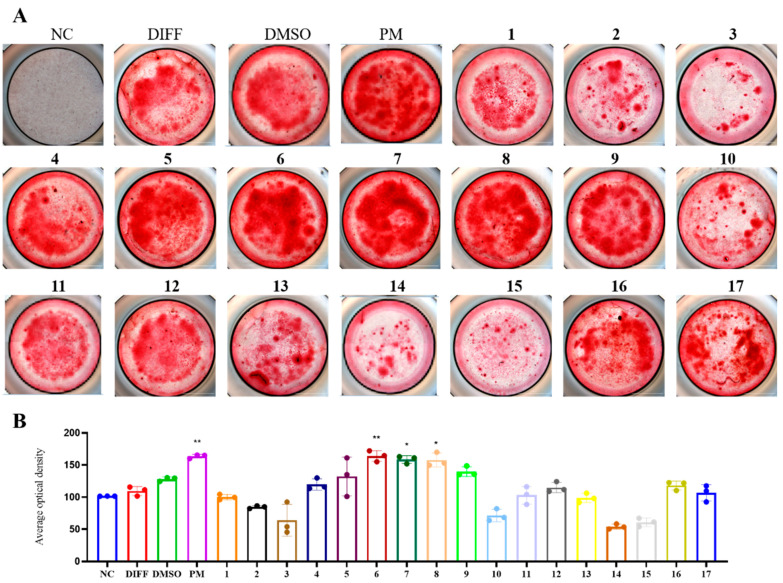
Preliminary screening results for the ability of compounds **1**–**17** to induce osteoblast activity among BMSCs. (**A**) Alizarin Red S staining. The BMSCs were cultures exposed to compounds **1**–**17** with osteogenic inducer. After 14 days, the cells were stained with Alizarin Red S and pictures were taken by Biotek cytation-5. The culture medium, osteogenesis differentiation medium (50 μg/mL ascorbic acid and 5 mM β-glycerophosphate), DMSO (0.1%), and purmorphamine (1 μM) were regarded as the negative control (NC), differentiation (DIFF), solvent (DMSO), and positive control (PM) groups, respectively. Scale bar = 1000 μm. (**B**) Quantification of Alizarin Red S staining based on average optical density. * *p* < 0.05, ** *p* < 0.01 vs. DMSO.

Intracellular calcium deposition at a later stage served as a significant evaluation indicator for osteogenic activity. Interestingly, compounds **6**, **7**, and **8** exerted a noticeable enhancing effect on osteoblast mineralization within BMSCs (Figure 8).

**Figure 9:**
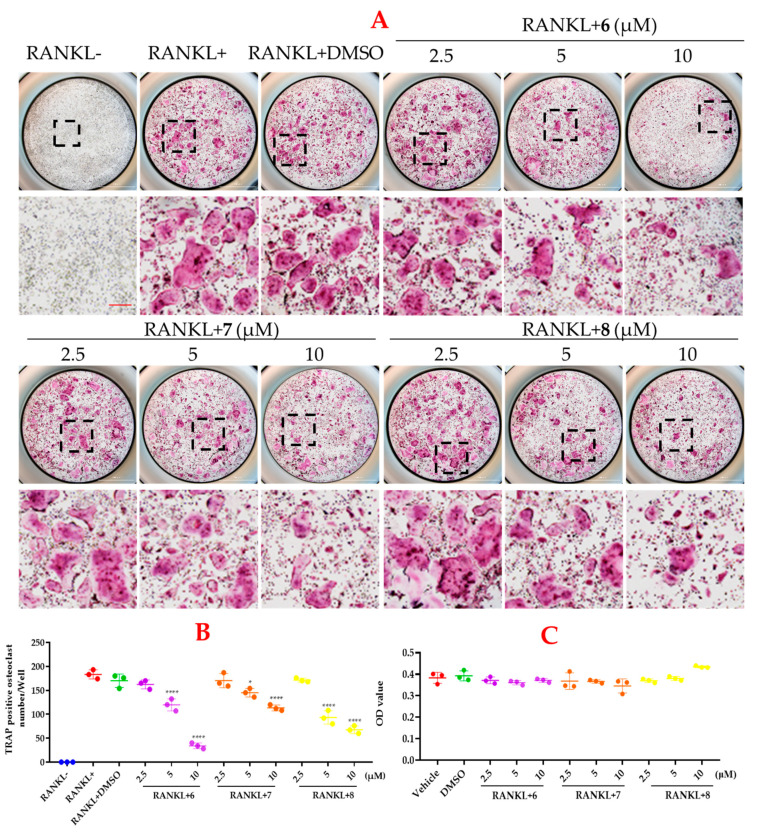
Compounds **6**, **7**, and **8** dose-dependently attenuated RANKL-activated osteoclastogenesis. (**A**) BMMs cultured with tested compounds (2.5 μM, 5.0 μM, and 10 μM) with the stimulation of 25 ng/mL RANKL (or not). After five days, these cells were fixed and stained with TRAP. (**B**) TRAP-positive multinucleated (there or more) cells were regarded as mature osteoclasts. Osteoclast numbers were quantified and analyzed (*n* = 3). * *p* < 0.05, **** *p* < 0.0001 vs. the RANKL+DMSO group. (**C**) BMM viability was measured by the CCK-8 assay. The culture medium and the solvent (0.1% DMSO) were regarded as Vehicle and DMSO groups, respectively.

Furthermore, bioactive compounds **6**, **7**, and **8** also exhibited a distinct inhibitory effect on osteoclast activity, as evidenced by a significant reduction in tartrate-resistant acid phosphatase (TRAP)-positive cells (Figure 9). These findings indicate that compounds **6**, **7**, and **8** not only facilitated osteoblast mineralization but also exerted a substantial dose-dependent inhibitory effect on RANKL-induced osteoclasts. 

## 3. Materials and Methods

### 3.1. General Experimental Procedures

NMR spectra of all compounds were recorded on a Bruker 400 MHz spectrometer (Bruker, Fällanden, Switzerland). Optical rotations were measured by an MCP 100 polarimeter (Anton Paar Trading Co., Ltd., Shanghai, China). The high-resolution electrospray ionization mass spectrometry (HRESIMS) results were acquired on a Xevo G2 Q-TOF mass spectrometer (Waters Corporation, Milford, MA, USA). ECD spectra were measured on a Chirascan spectrometer (Applied Photophysics, Surrey, UK). The semi-preparative high-performance liquid chromatography (semi-prep. HPLC) was performed on an Agilent instrument (1260) (Agilent Technologies, San Diego, CA, USA) with a semi-preparative chromatographic column (COSMOSIL 5 C18-MS-II, Nacalai Tesque, Japan). Column chromatography (CC) was performed on silica gel (Qingdao Marine Chemistry Co., Ltd., Qingdao, China), Sephadex LH-20 (Amersham Pharmacia Biotech AB, Uppsala, Sweden), and octadecyl silyl (ODS) (Daiso Chemical Co., Ltd., Osaka, Japan). Preparative thin-layer chromatography (Prep. TLC) was performed with silica gel precoated plates (Qingdao Marine Chemistry Co., Ltd., Qingdao, China).

### 3.2. Fungal Identification, Fermentation, and Extraction

The fungal strain W17 was isolated from a deep-sea sediment sample of the western Pacific Ocean at the depth of 5278 m. It was identified to be *Penicillium citrinum* as the 18S rRNA gene sequence alignment (OR398934) demonstrated that it showed great similarity (99.8%) to *Penicillium citrinum* NRRL 1841 (GenBank accession number NR_121224.1). The strain was preserved at the Key Laboratory of Marine Genetic Resources, Third Institute of Oceanography, Ministry of Natural Resources (Xiamen, China). The microbial strain was cultivated on a PDA plate medium at 25 °C for 3 days and the colony was inoculated into 250 mL Erlenmeyer flasks containing 50 mL PDB medium. Then, it was cultured in a rotary shaker (130 rpm) at 25 °C for 3 days as spore medium. After 3 days, the spore solution was inoculated in 120 Erlenmeyer flasks (1 L) with each containing 400 mL tap water, 80 g potato power, 8 g glucose, and 6 g marine salt. The fermentation was performed in a 130 rpm rotary shaker at 25 °C. After 7 days, the fermentation broth was extracted with EtOAc three times and concentrated under reduced pressure to give a crude extract (36.5 g).

### 3.3. Isolation and Purification

The crude extract (36.5 g) was separated into seven fractions (Fr.1−Fr.7) via CC over silica gel using a gradient of CH_2_Cl_2_−MeOH (100%→75%). Fraction Fr.4 (3.6 g) was subsequently separated by CC over ODS (MeOH-H_2_O, 5%→100%), Sephadex LH-20 (MeOH), and semi-prep. HPLC with MeOH-H_2_O (40%→100%) to yield **1** (5.7 mg) and **4** (15.4 mg). Fraction Fr.7 (1.5 g) was separated by CC over Sephadex LH-20 (MeOH), silica gel (CH_2_Cl_2_−MeOH, 10:1), and semi-prep. HPLC with MeOH−H_2_O (30%→60%) to yield **2** (1.0 mg), **3** (1.0 mg), and **6** (45.9 mg). Compound **11** (43.0 mg) was purified from Fr.1 (5.0 g) by recrystallization in MeOH, while compounds **14** (14.0 mg) and **16** (49.0 mg) were isolated by CC over ODS (50%→100%), Sephadex LH-20 (MeOH), and prep. TLC (CH_2_Cl_2_-MeOH, 50:1). Fraction Fr.2 (2.0 g) was subjected to ODS (10%→100%) and Sephadex LH-20 (MeOH) to yield compound **12** (69.0 mg). Fraction Fr.3 was subjected to CC on ODS (10%→100%) and Sephadex LH-20 (MeOH). Final purification by semi-prep. HPLC (MeOH-H_2_O, 60%→90%) afforded **9** (1.6 mg) and **10** (3.7 mg). Fraction Fr.5 (4.6 g) was separated by CC over ODS (10%→100%), Sephadex LH-20 (MeOH), and semi-prep. HPLC (MeOH-H_2_O, 30%→60%) to give **5** (124.0 mg), **13** (7.0 mg), **15** (2.1 mg), and **17** (190.0 mg). Compounds **7** (15.2 mg) and **8** (22.4 mg) were obtained by CC over ODS (10%→100%), Sephadex LH-20 (MeOH), and semi-prep. HPLC (MeOH−H_2_O, 30%→60%) from Fr.6 (2.0 g).

Penidihydrocitrinin A (**1**): yellow amorphous powder; [*α*${]}_{D}^{25}$ +28.0 (*c* 0.10, MeOH); UV (MeOH) *λ*max (logε) 214 (3.16), 244 (2.64), 252 (2.65), 280 (1.85), 319 (2.16) nm; CD (MeOH) (Δε) 229 (+5.00), 233 (+0.53), 286 (−2.20), 330 (+0.42) nm; ^1^H and ^13^C NMR data, see Table 1; HRESIMS *m*/*z* 337.1309 [M − H]^−^ (calcd for C_17_H_21_O_7_ 337.1287).

Penidihydrocitrinin B (**2**): colorless oil; [*α*${]}_{D}^{25}$ +25.0 (*c* 0.10, MeOH); UV (MeOH) *λ*max (logε) 204 (2.78), 214 (2.84), 242 (2.28), 253 (2.32), 276 (1.40), 319 (1.90) nm; CD (MeOH) (Δε) 228 (+1.82), 282 (−0.34), 322 (+0.18) nm; ^1^H and ^13^C NMR data, see Table 1; HRESIMS *m*/*z* 337.1348 [M − H]^−^(calcd for C_17_H_21_O_7_ 337.1287).

Penidihydrocitrinin C (**3**): colorless oil; [*α*${]}_{D}^{25}$ +40.0 (*c* 0.10, MeOH); UV (MeOH) *λ*max (logε) 205 (2.71), 213 (2.72), 253 (2.24), 275 (1.39), 320 (1.83) nm; CD (MeOH) (Δε) 228 (+0.94), 260 (+0.26), 287 (+0.54) nm; ^1^H and ^13^C NMR data, see Table 1; HRESIMS *m*/*z* 337.1327 [M − H]^−^ (calcd for C_17_H_21_O_7_, 337.1287).

### 3.4. ECD Calculation

As reported previously [16], the conformational analysis was first conducted via random searching stochastically using the MMFF94 force field. All conformers were consecutively optimized at the PM6 and HF/6-31G(d) levels. Dominative conformers were further optimized at the B3LYP/6-31G(d) level in the gas phase. The optimized conformers possessed no imaginary frequencies and were true local minima. ECD calculations were conducted at the B3LYP/6-311G(d,p) level in MeOH with the IEFPCM model using time-dependent density functional theory (TD-DFT). The ECD spectrum was simulated by overlapping Gaussian functions for each transition.

### 3.5. BV-2 Cell Culture and Compound Treatment

BV-2 cell culture and compound treatment were carried out as previously reported [43,44]. Briefly, BV-2 microglial cells were cultured in DMEM supplemented with 10% fetal bovine serum (Thermofisher, Shanghai, China) and antibiotics (100 units/mL of penicillin and 100 μg/mL of streptomycin) in a humidified 5% CO_2_ incubator at 37 °C. Cells were seeded into 24-well plates at a density of 2 × 10^4^ cells per well and allowed to adhere overnight. On the subsequent day, the cells were treated with freshly prepared culture medium containing the specified concentrations of the investigated compounds for a duration of 30 min before exposure to LPS (1 μg/mL). A control group was treated with a vehicle solution (DMSO, 0.1%).

### 3.6. Quantification of Nitrite Levels

The concentration of nitrite present in the culture medium was assessed using the Griess Reagent Kit (Thermo Fisher, Shanghai, China). Briefly, 75 μL of cell culture supernatants was mixed with an equal volume of the Griess Reagent Kit and allowed to react for 30 min at room temperature. The absorbance of the resulting diazonium compound was measured at a wavelength of 560 nm. The concentration of nitrite production was calculated based on the nitrite standard solution.

### 3.7. Cell Extraction and Culture

The bone mesenchymal stem cells (BMSCs) and bone marrow monocytes (BMMs) were flushed from the femur of C57BL/6J mice aged 3 weeks and 6-week-old C57BL/6 mice, respectively, with the methods as previously described [16]. In brief, the BMSCs were carefully removed from the animals and cultured in α-MEM and induced with supplemented complete α-MEM (10% *v/v* FBS, 1% *v/v* penicillin/streptomycin (P/S)). The BMSCs were induced with 2 mM β-glycerophosphate and 50 μg/mL ascorbate, of which half were changed twice a week. The BMMs were cultured in complete α-MEM (10% *v/v* FBS, 1% *v/v* P/S, and 25 ng/mL M-CSF).

### 3.8. CCK-8 Assay

The in vitro cytotoxic bioassay was conducted using the CCK-8 method according to the previously reported protocols [16]. The BMSCs or BMMs were treated with or without compounds. After 48 h of culture, cells were treated with CCK-8 solution for 2 h before being scanned with a multimode scanner at 450 nm (Biotek, Winooski, VT, USA).

### 3.9. Osteoblast Differentiation and Mineralization

The BMSCs were digested and planted at a density of 2 × 10^4^ cells/well into 96-well plates and cultivated overnight. Ascorbic acid (50 μg/mL) and β-glycerophosphate (5 mM) were added into the culture medium for osteogenic assay. The culture medium was changed every other day. AR S staining was performed after 14 days of differentiation. Alizarin Red staining was used to check the calcification conditions in cultures. Cells were fixed with 10% neutral-buffered formalin for 30 min and then 2% Alizarin Red S was used to incubate cells for 2 min at room temperature. Then, pictures were taken of the cells by a Biotek cytation-5.

### 3.10. Osteoclast Differentiation

BMMs were cultured for 7 days in the presence of M-CSF (25 ng/mL) and RANKL (25 ng/mL) for differentiation into mature osteoclasts. Media were refreshed every 2 days. For tartrate-resistant acid phosphatase (TRAP) staining, cells were fixed with 4% paraformaldehyde (PFA) and stained for TRAP activity. Under a microscope (Biotek cytation-5, USA), TRAP-positive multinucleated cells with three nuclei were counted as osteoclasts. 

## 4. Conclusions

In summary, from the deep-sea-derived fungus *Penicillium citrinum* W17, 17 compounds were obtained. Penidihydrocitrinins A–C (**1**–**3**) are three novel citrinin and diacetyl adducts, which greatly enrich the chemical diversity of *Penicillium* species. Compound **14** displayed potent anti-inflammatory activity. Meanwhile, **6**, **7**, and **8** not only significantly promoted the osteoblast mineralization but also inhibited osteoclasts, providing potent drug leads for anti-osteoporosis.

## Figures and Tables

**Table 1:** ^1^H (400 Hz) and ^13^C (100 Hz) NMR spectroscopic data of **1**–**3** (*δ* in ppm, *J* in Hz within parentheses).

No.	1 ^a^		2 ^b^		3 ^b^	
	*δ*_C_	*δ*_H_	*δ*_C_	*δ*_H_	*δ*_C_	*δ*_H_
1	75.4 CH	5.17 s	64.8 CH	5.08 (d, 9.7)	64.8 CH	5.08 (d, 9.2)
3	74.0 CH	3.98 (qd, 6.9, 1.8)	71.7 CH	3.82 (qd, 6.6, 1.5)	71.7 CH	3.82 (qd, 6.6, 1.5)
4	36.9 CH	2.62 (qd, 6.7, 1.8)	35.1 CH	2.52 m	35.2 CH	2.51 m
5	114.0 C		109.5 C		109.6 C	
6	160.0 C		158.5 C		158.5 C	
7	102.2 C		101.8 C		101.8 C	
8	156.7 C		155.8 C		155.8 C	
9	110.3 C		110.7 C		110.9 C	
10	143.8 C		139.7 C		139.8 C	
11	18.3 CH_3_	1.11 (d, 6.4)	18.2 CH_3_	1.01 (d, 6.5)	18.3 CH_3_	0.99 (d, 6.5)
12	20.1 CH_3_	1.28 (d, 6.9)	20.1 CH_3_	1.17 (d, 6.8)	20.2 CH_3_	1.17 (d, 7.2)
13	10.2 CH_3_	2.04 s	9.5 CH_3_	1.93 s	9.6 CH_3_	1.92 s
14	178.2 C		175.6 C		175.7 C	
15	83.2 C		43.1 CH_2_	2.66 m 3.23 m	43.0 CH_2_	2.72 m 3.16 m
16	211.3 C		211.9 C		212.1 C	
17	25.0 CH_3_	2.30 s	72.2 CH	4.11 m	72.8 CH	4.02 m
18	20.7 CH_3_	1.10 s	19.2 CH_3_	1.21 (d, 7.0)	19.0 CH_3_	1.15 (d, 7.2)
6-OH				14.64 s		14.62 s
8-OH				15.16 s		15.13 s

^a^ Recorded in CD_3_OD. ^b^ Recorded in DMSO-*d*_6_.

## Data Availability

The data presented in this study are available in Supplementary Materials.

## Supplementary Materials

The following are available online at https://www.mdpi.com/article/10.3390/md21100538/s1, Figures S1–S21: One-dimensional and two-dimensional NMR spectra along with HRESIMS spectra of compounds **1**–**3**.
