# Supplementary material for: Penidihydrocitrinins A–C: New Polyketides from the Deep-Sea-Derived Penicillium citrinum W17 and Their Anti-Inflammatory and Anti-Osteoporotic Bioactivities
Source: Mar Drugs. 2023 Oct 14;21(10):538. doi: 10.3390/md21100538 (PMC10608093; doi:10.3390/md21100538)
Supplement: Supplementary file 1 [file marinedrugs-21-00538-s001.zip › marinedrugs-2654070-supplementary.pdf]

Supporting Information

Penidihydrocitrinins A–C: New Polyketides from the Deep-Sea-Derived *Penicillium citrinum* W17 and Their Anti-Inflammatory and Anti-osteoporotic Bioactivities

Yong Zhang [1,†], Chun-Lan Xie [1,2,†], Yuan Wang [1], Xi-Wen He [1], Ming-Min Xie [1], You Li [1], Kai Zhang [1], Zheng-Biao Zou [1], Long-He Yang [1,*], Ren Xu [2,*], and Xian-Wen Yang [1,*]

[1] Key Laboratory of Marine Genetic Resources, Technical Innovation Center for Utilization of Marine Biological Resources, Third Institute of Oceanography, Ministry of Natural Resources, 184 Daxue Road, Xiamen 361005, China

[2] School of Medicine, Xiamen University, South Xiangan Road, Xiamen 361005, China

[*] Correspondence: yangxianwen@tio.org.cn (X.-W.Y.); xuren526@xmu.edu.cn (R.X.); longheyang@tio.org.cn (L.Y.); Tel.: +86-592-219-5319 (X.-W.Y.); +86-592-288-0577 (R.X.); +86-592-219-5319 (L.Y.)

[†] These authors contributed equally to this work.

Content

Figure S1. The HR-ESI-MS of compound **1**.
Figure S2. The ^{1}H NMR spectrum of compound **1** in CD_3OD.
Figure S3. The ^{13}C NMR spectrum of compound **1** in CD_3OD.
Figure S4. The HSQC spectrum of compound **1** in CD_3OD.
Figure S5. The ^{1}H-^{1}H COSY spectrum of compound **1** in CD_3OD.
Figure S6. The HMBC spectrum of compound **1** in CD_3OD.
Figure S7. The NOESY spectrum of compound **1** in CD_3OD.
Figure S8. The HR-ESI-MS of compound **2**.
Figure S9. The ^{1}H NMR spectrum of compound **2** in DMSO-d_6.
Figure S10. The ^{13}C NMR spectrum of compound **2** in DMSO-d_6.
Figure S11. The HSQC spectrum of compound **2** in DMSO-d_6.
Figure S12. The ^{1}H-^{1}H COSY spectrum of compound **2** in DMSO-d_6.
Figure S13. The HMBC spectrum of compound **2** in DMSO-d_6.
Figure S14. The NOESY spectrum of compound **2** in DMSO-d_6.
Figure S15. The HR-ESI-MS of compound **3**.
Figure S16. The ^{1}H NMR spectrum of compound **3** in DMSO-d_6.
Figure S17. The ^{13}C NMR spectrum of compound **3** in DMSO-d_6.
Figure S18. The HSQC spectrum of compound **3** in DMSO-d_6.
Figure S19. The ^{1}H-^{1}H COSY spectrum of compound **3** in DMSO-d_6.
Figure S20. The HMBC spectrum of compound **3** in DMSO-d_6.
Figure S21. The NOESY spectrum of compound **3** in DMSO-d_6.

Elemental Composition Report

Tolerance = 5.0 mDa / DBE: min = -1.5, max = 50.0
Element prediction: Off
Number of isotope peaks used for i-FIT = 3

Monoisotopic Mass, Even Electron Ions
105 formula(e) evaluated with 3 results within limits (up to 50 best isotopic matches for each mass)
Elements Used:
C: 0-35 H: 0-50 O: 0-15 23Na: 0-1
ZYA 5-2-N 86 (0.347) Cm (75:109)
1: TOF MS ES-

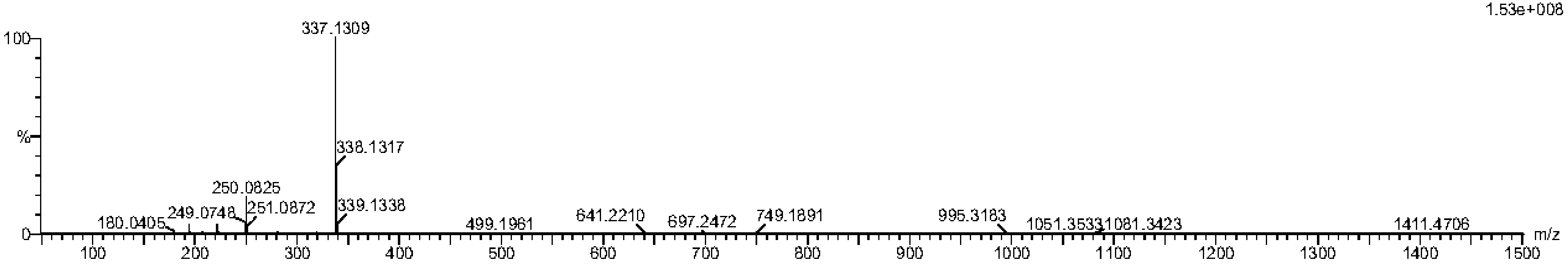

Minimum:	80.00				-1.5				
Maximum:	100.00		5.0	10.0	50.0				
Mass	RA	Calc. Mass	mDa	PPM	DBE	i-FIT	Norm	Conf(%)	Formula
337.1309	100.00	337.1287	2.2	6.5	7.5	2059.9	0.584	55.77	C17 H21 O7
		337.1263	4.6	13.6	4.5	2060.5	1.170	31.05	C15 H22 O7 23Na
		337.1346	-3.7	-11.0	-1.5	2061.3	2.026	13.18	C10 H25 O12

Figure S1. The HR-ESI-MS of compound **1**.

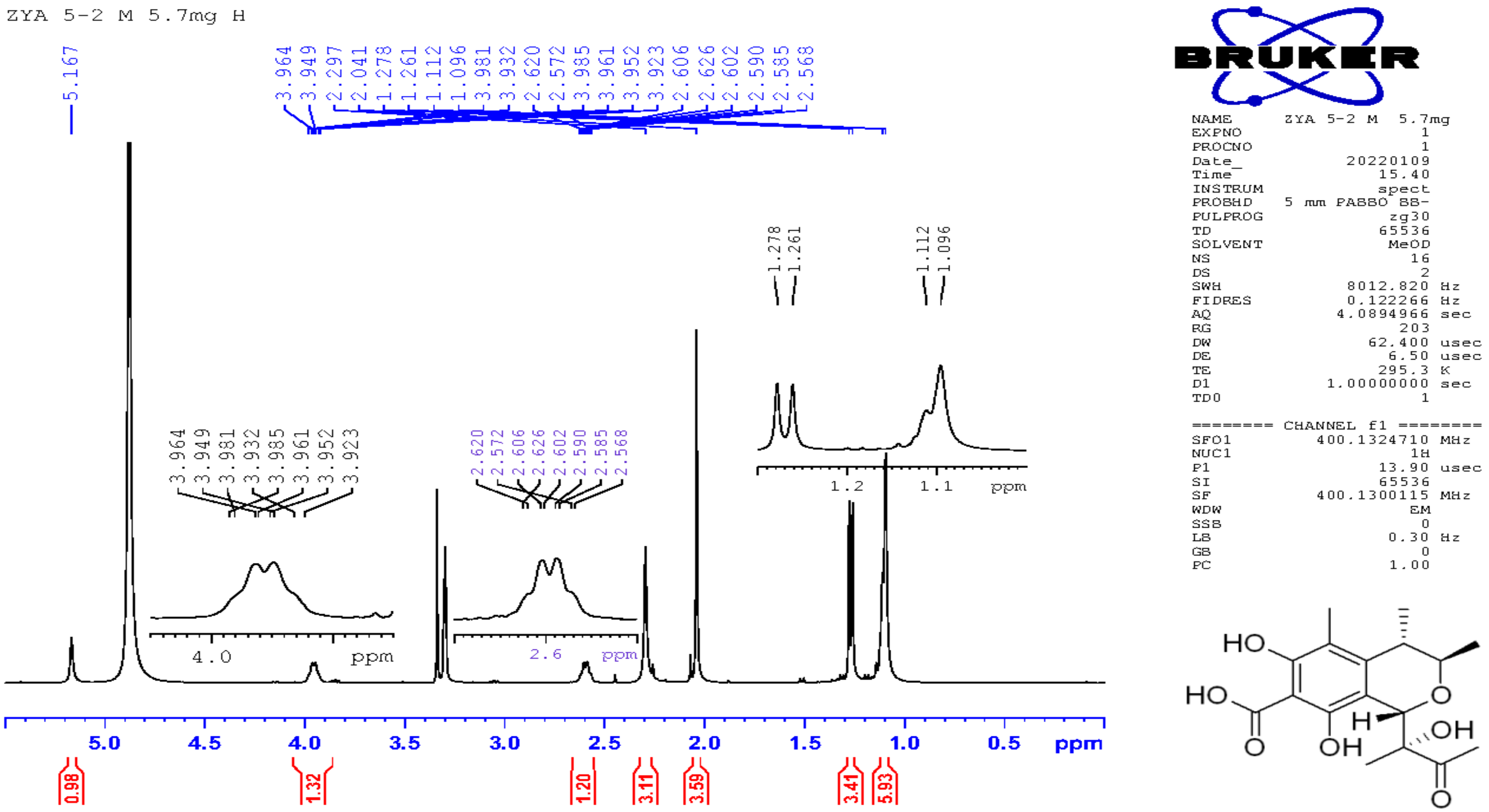

Figure S2. The ^{1}H NMR spectrum of compound **1** in CD_3OD.

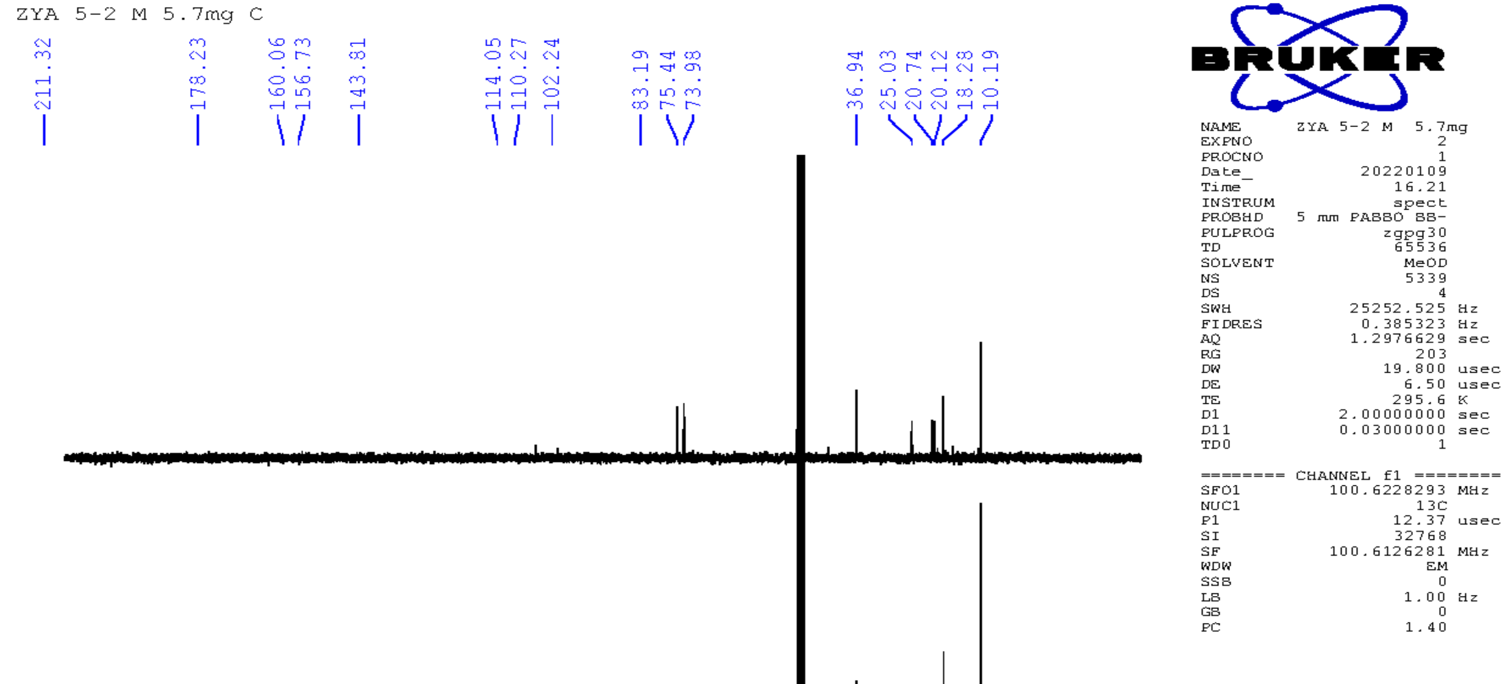

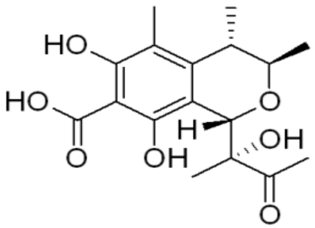

Figure S3. The ^{13}C NMR spectrum of compound **1** in CD_3OD.

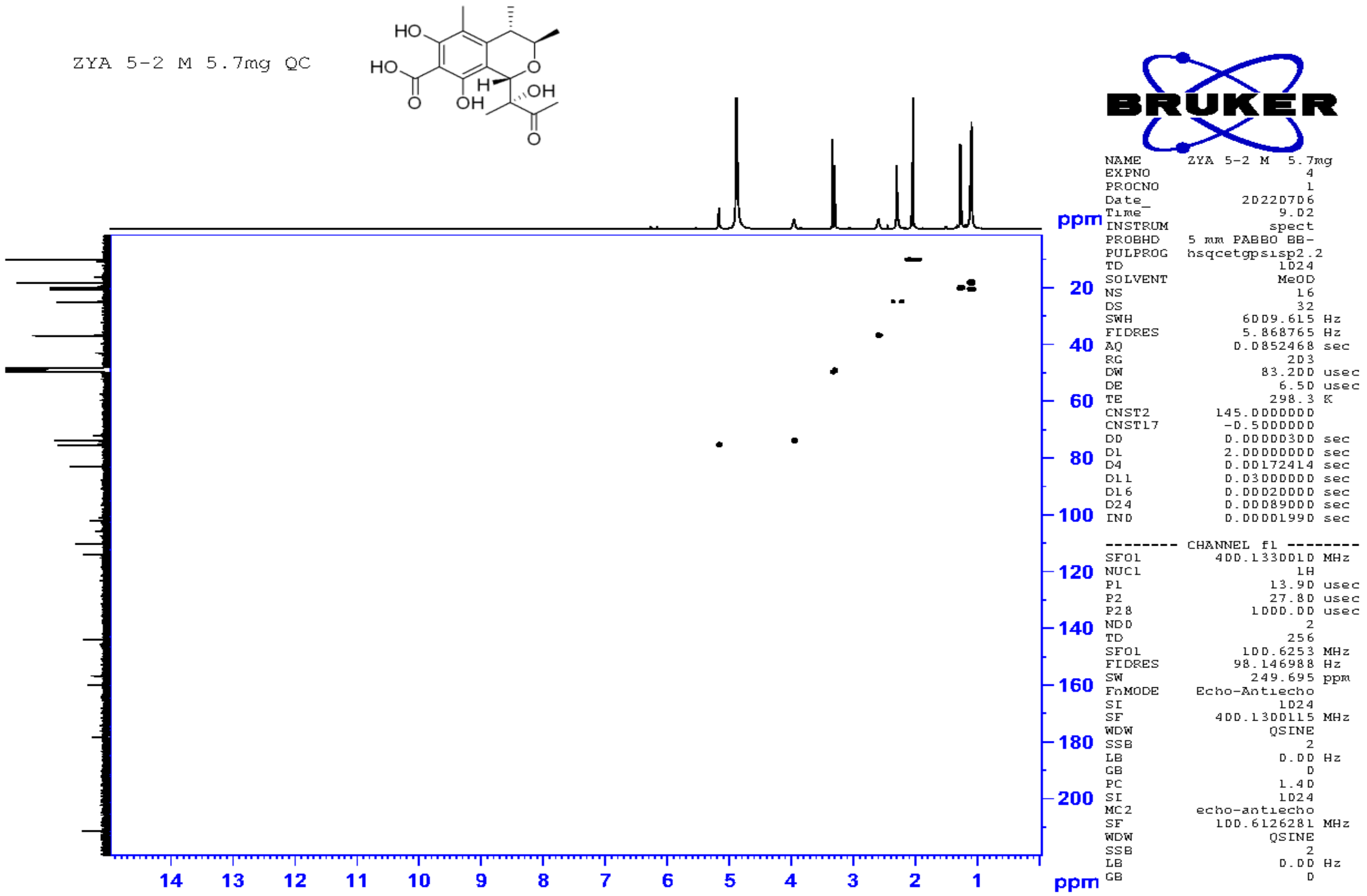

Figure S4. The HSQC spectrum of compound **1** in CD_3OD.

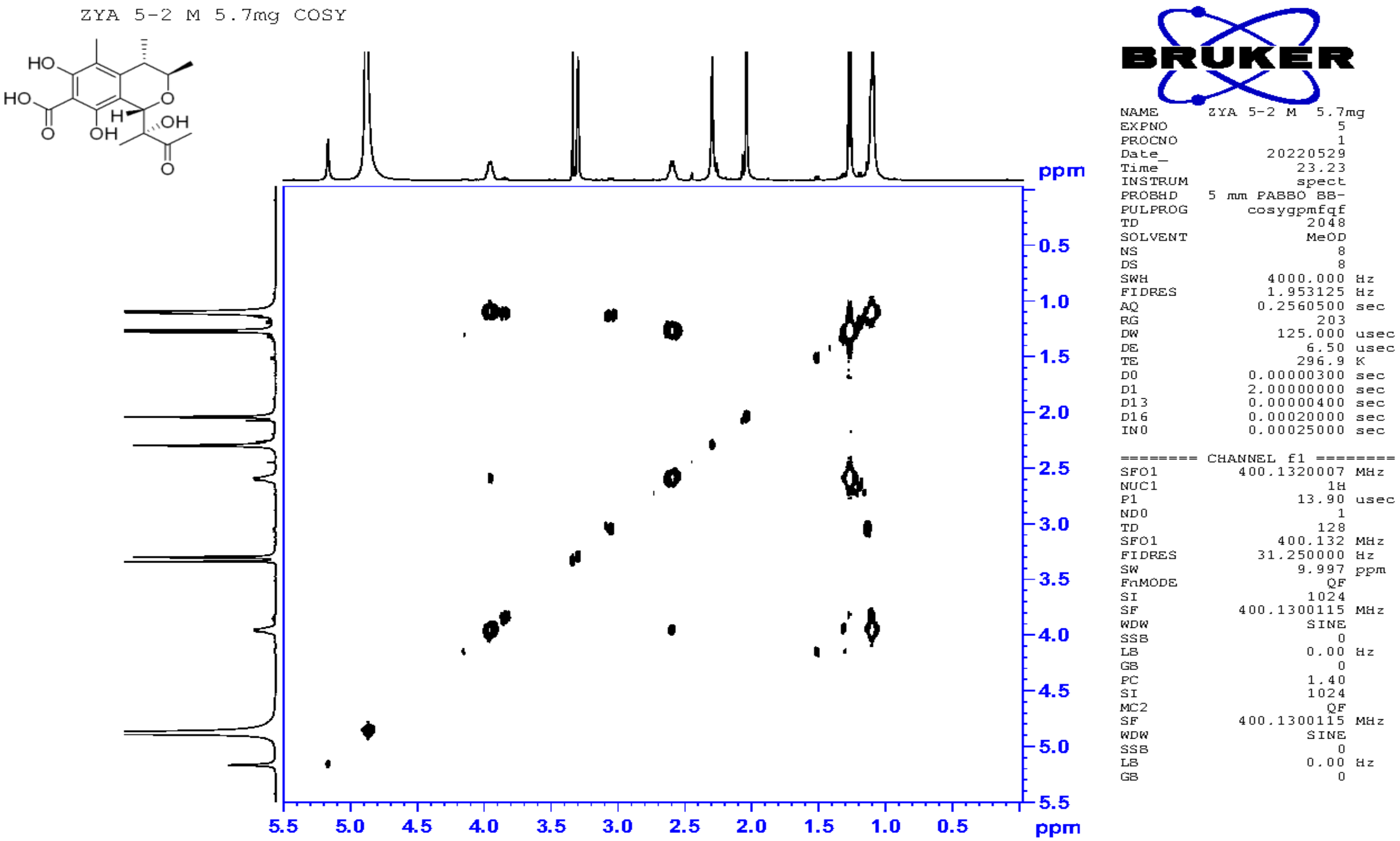

Figure S5. The ^{1}H-^{1}H COSY spectrum of compound **1** in CD_3OD.

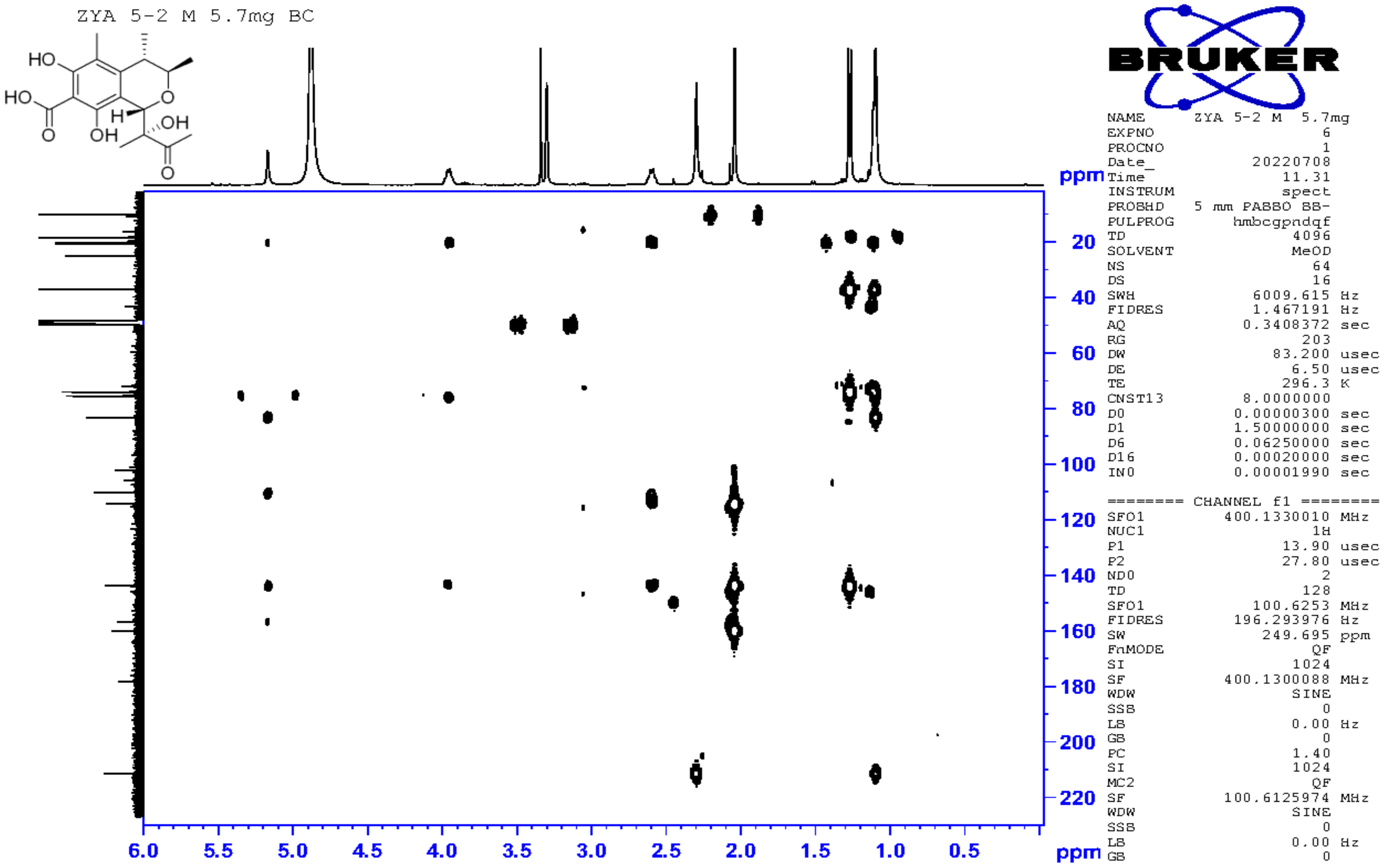

Figure S6. The HMBC spectrum of compound **1** in CD_3OD.

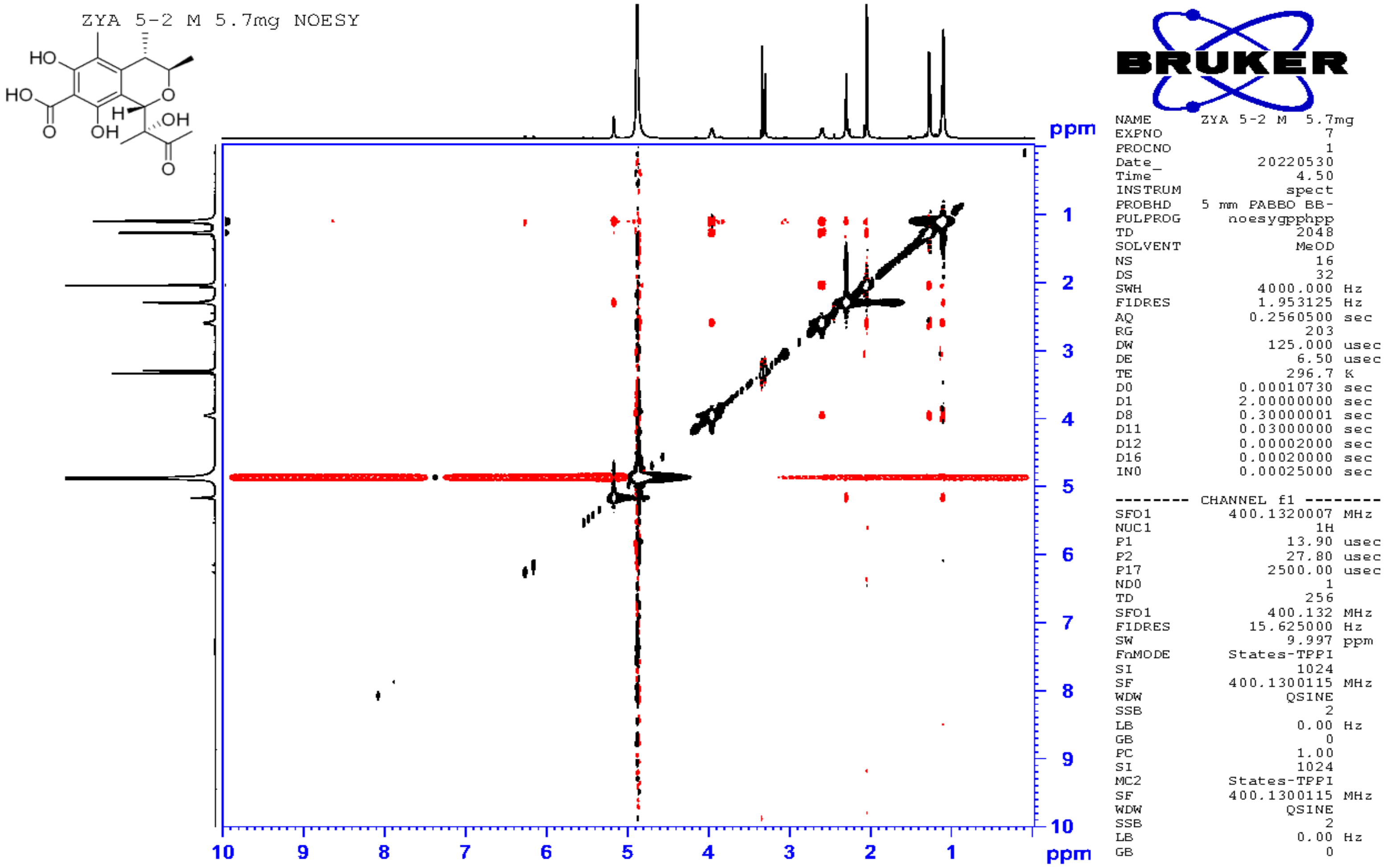

Figure S7. The NOESY spectrum of compound **1** in CD_3OD.

Tolerance = 50.0 PPM / DBE: min = -1.5, max = 50.0
Element prediction: Off
Number of isotope peaks used for i-FIT = 3

Monoisotopic Mass, Even Electron Ions
83 formula(e) evaluated with 8 results within limits (up to 50 best isotopic matches for each mass)
Elements Used:
C: 5-35 H: 0-60 O: 0-10 23Na: 0-1
ZYA 8-3-N 70 (0.290) Cm (70:71)
1: TOF MS ES-

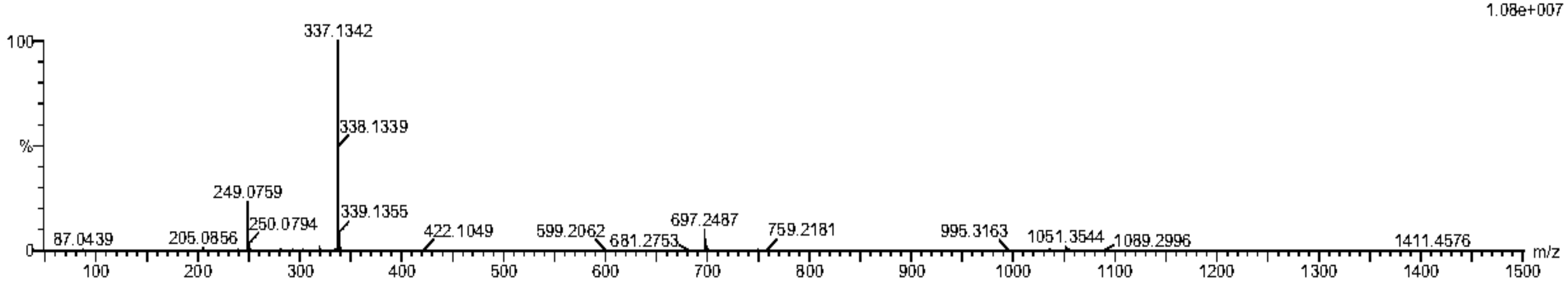

Minimum:	80.00				-1.5				
Maximum:	100.00		50.0	50.0	50.0				

Mass	RA	Calc. Mass	mDa	PPM	DBE	i-FIT	Norm	Conf(%)	Formula
337.1342	100.00	337.1229	11.3	33.5	16.5	1296.8	1.570	20.81	C24 H17 O2
		337.1440	-9.8	-29.1	11.5	1297.1	1.848	15.75	C21 H21 O4
		337.1204	13.8	40.9	13.5	1297.3	1.998	13.56	C22 H18 O2 23Na
		337.1287	5.5	16.3	7.5	1297.4	2.139	11.78	C17 H21 O7
		337.1416	-7.4	-21.9	8.5	1297.4	2.151	11.64	C19 H22 O4 23Na
		337.1499	-15.7	-46.6	2.5	1297.7	2.380	9.26	C14 H25 O9
		337.1263	7.9	23.4	4.5	1297.7	2.381	9.25	C15 H22 O7 23Na
		337.1475	-13.3	-39.5	-0.5	1297.8	2.531	7.96	C12 H26 O9 23Na

Figure S8. The HR-ESI-MS of compound **2**.

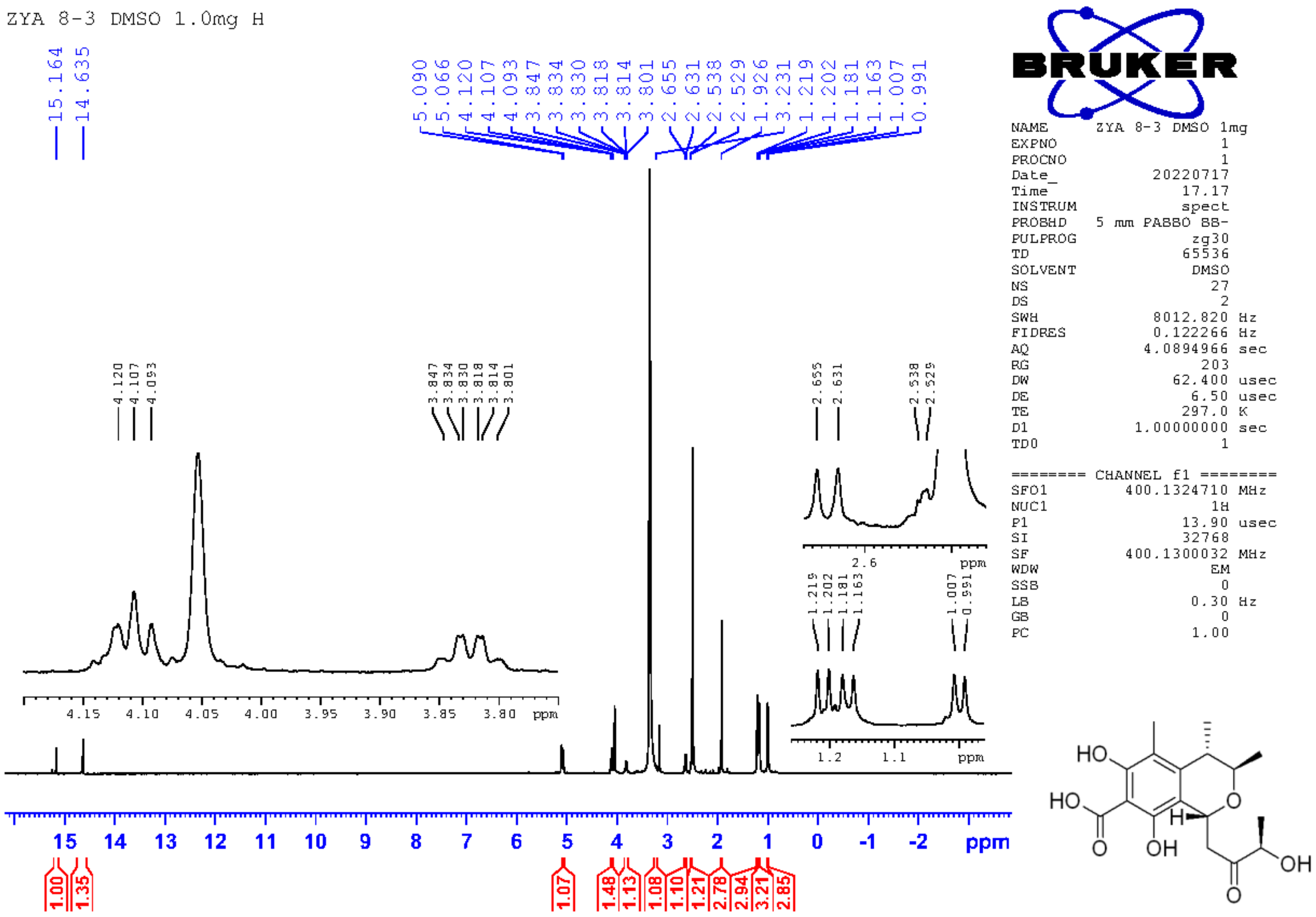

Figure S9. The ^{1}H NMR spectrum of compound **2** in DMSO-d_6.

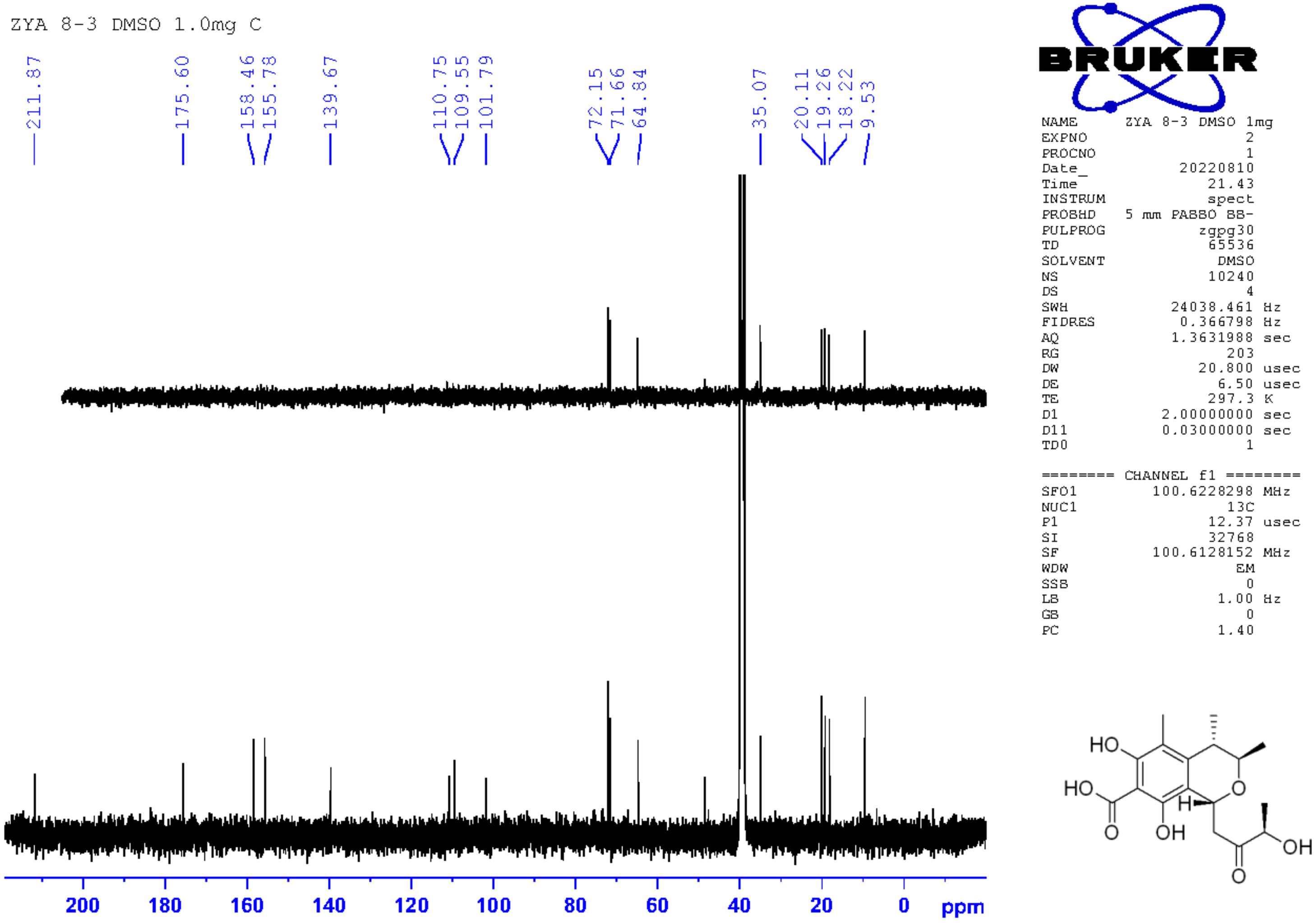

Figure S10. The ^{13}C NMR spectrum of compound **2** in DMSO-d_6.

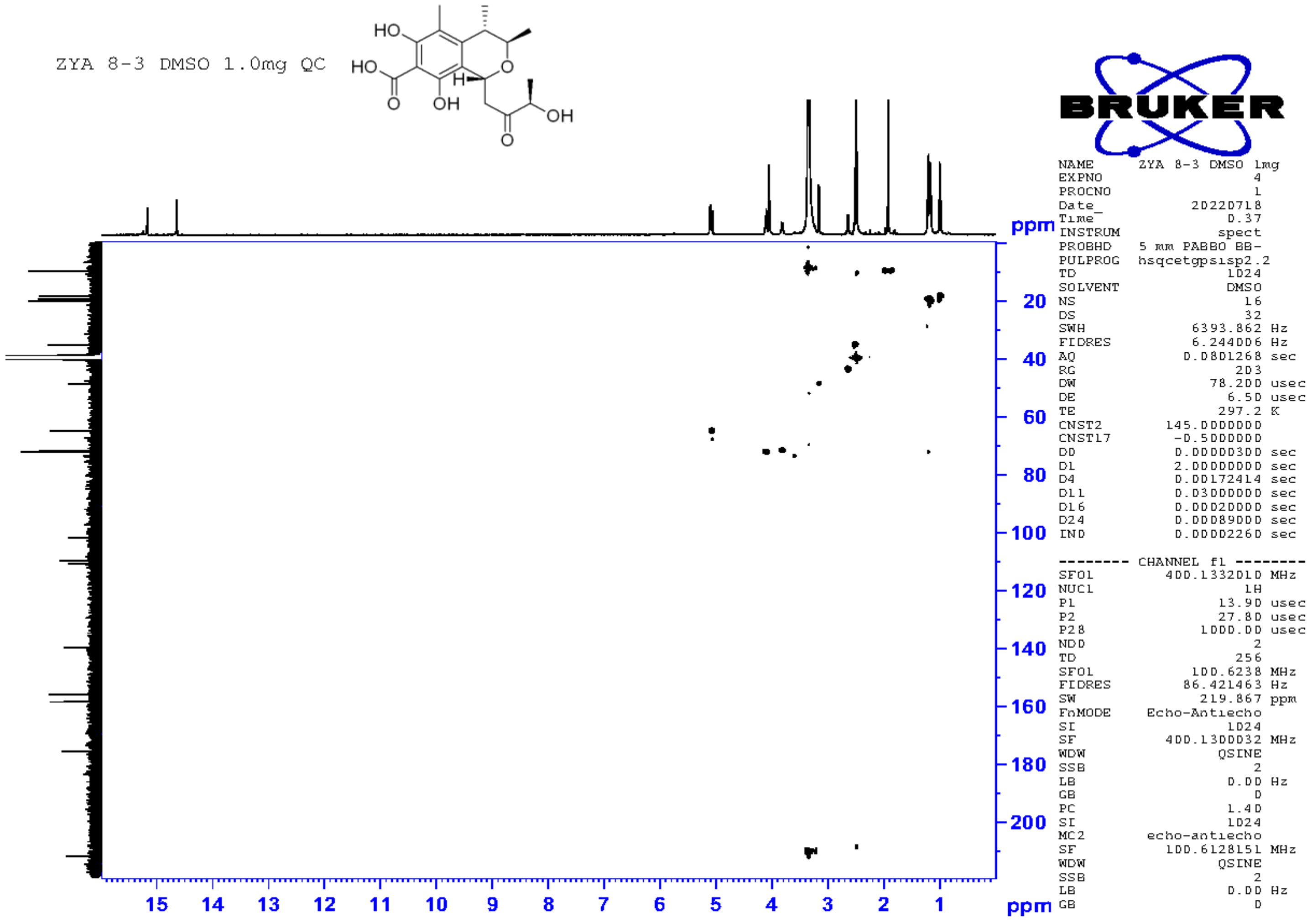

Figure S11. The HSQC spectrum of compound **2** in DMSO-d_6.

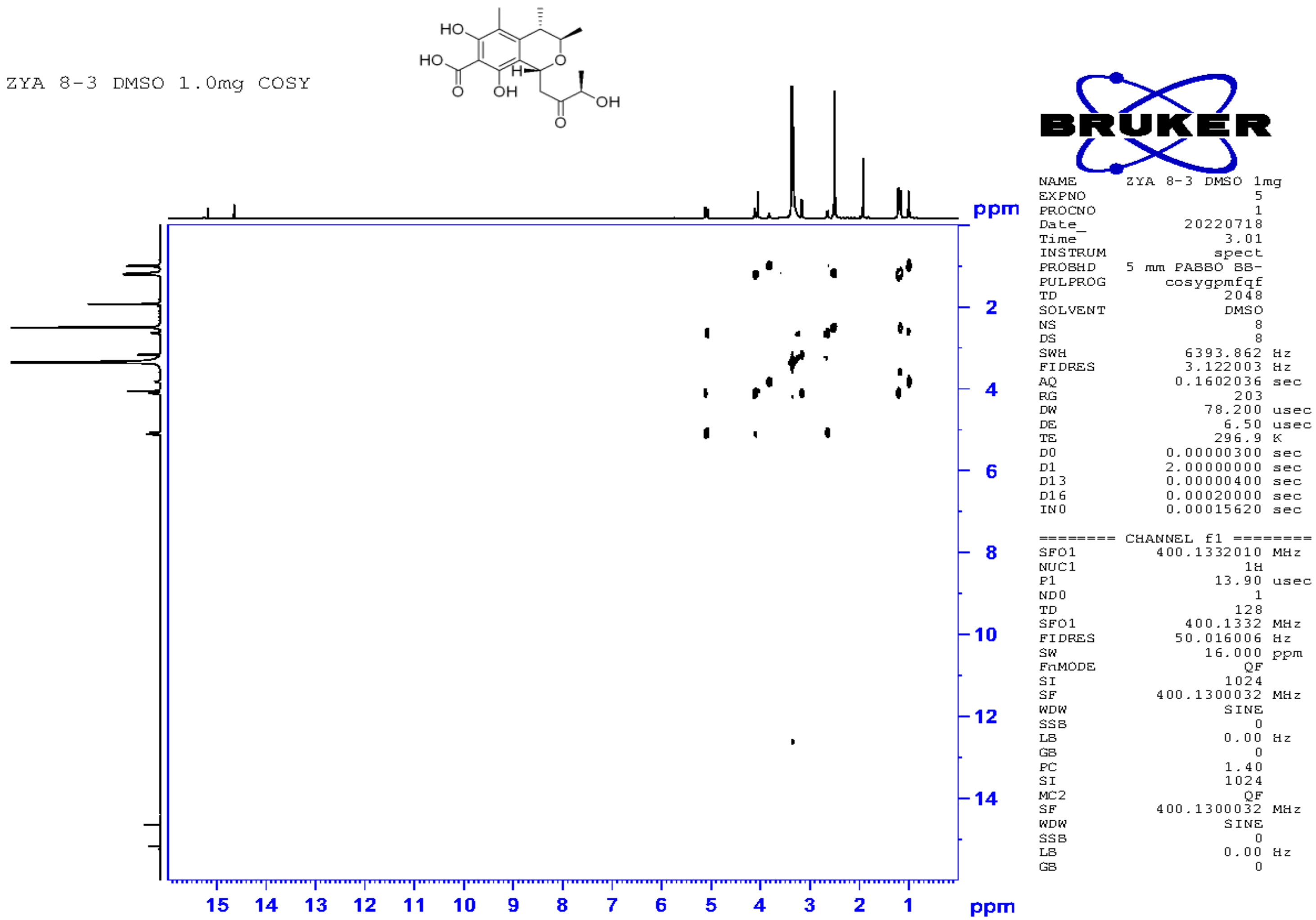

Figure S12. The ^{1}H-^{1}H COSY spectrum of compound **2** in DMSO-d_6.

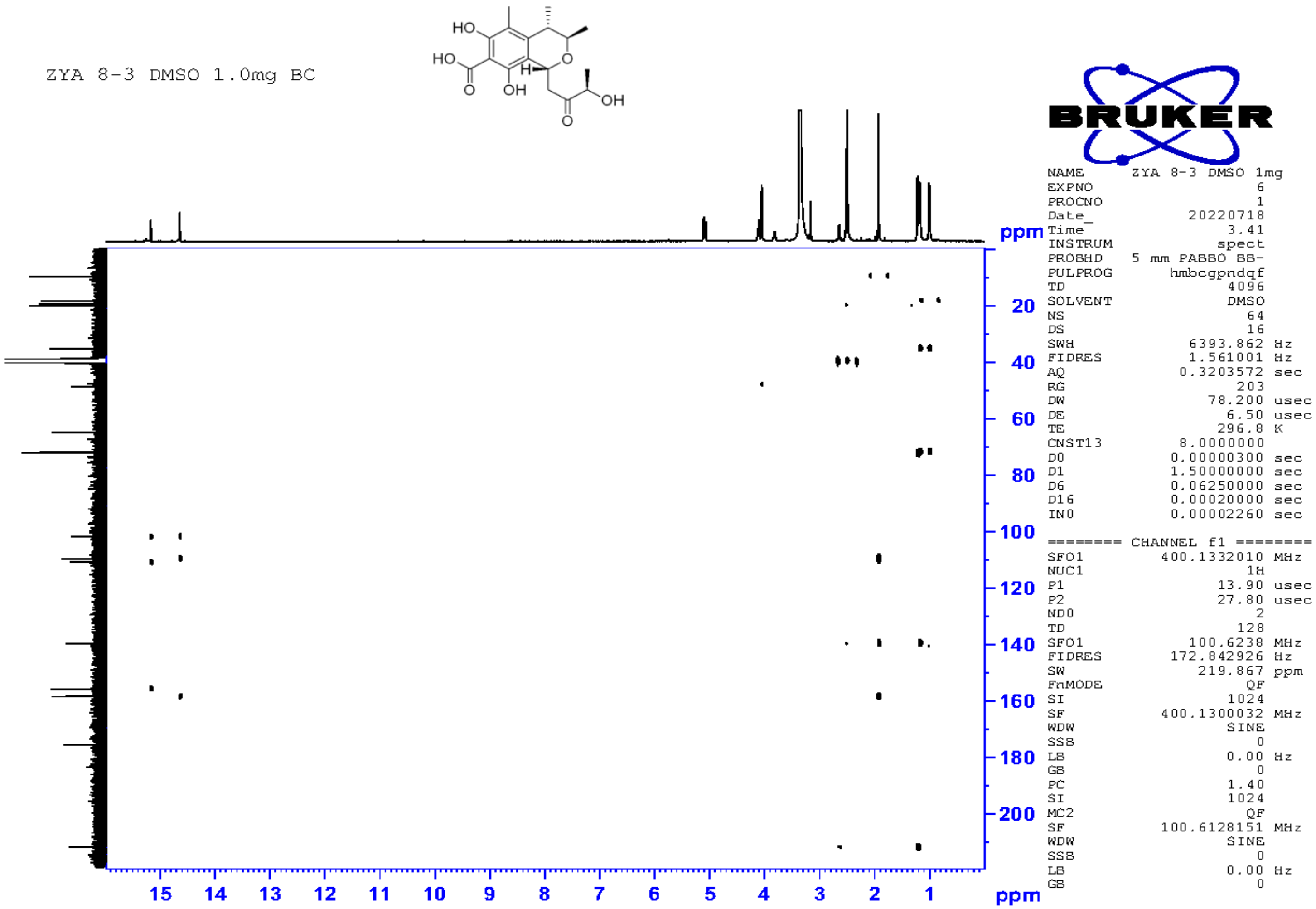

Figure S13. The HMBC spectrum of compound **2** in DMSO-d_6.

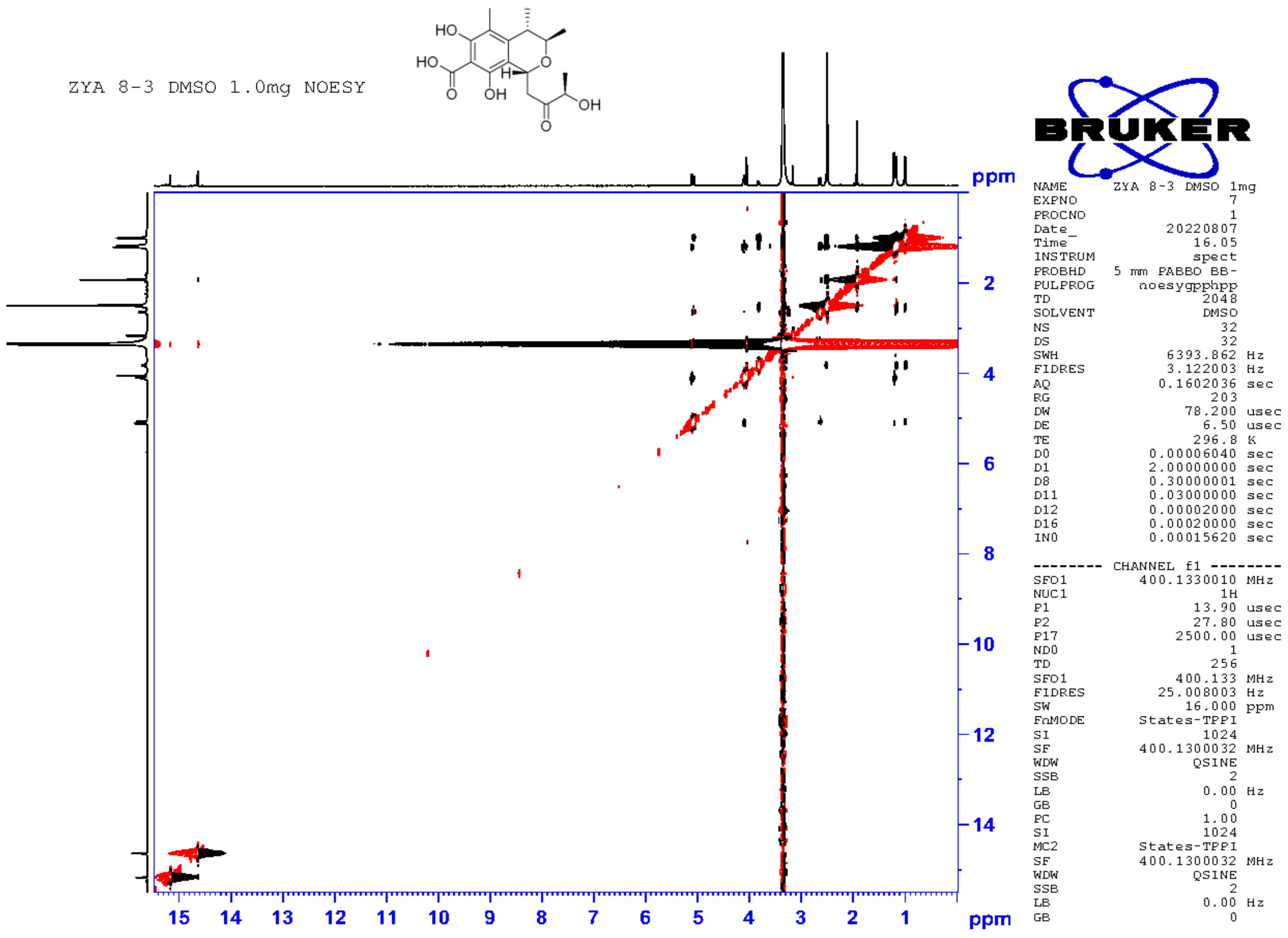

Figure S14. The NOESY spectrum of compound **2** in DMSO-d_6.

Elemental Composition Report

Tolerance = 5.0 mDa / DBE: min = -1.5, max = 50.0
Element prediction: Off
Number of isotope peaks used for i-FIT = 3

Monoisotopic Mass, Even Electron Ions
105 formula(e) evaluated with 2 results within limits (up to 50 best isotopic matches for each mass)
Elements Used:
C: 0-35 H: 0-50 O: 0-15 23Na: 0-1
ZYA 8-4-N 70 (0.290) Cm (63:89)
1: TOF MS ES-

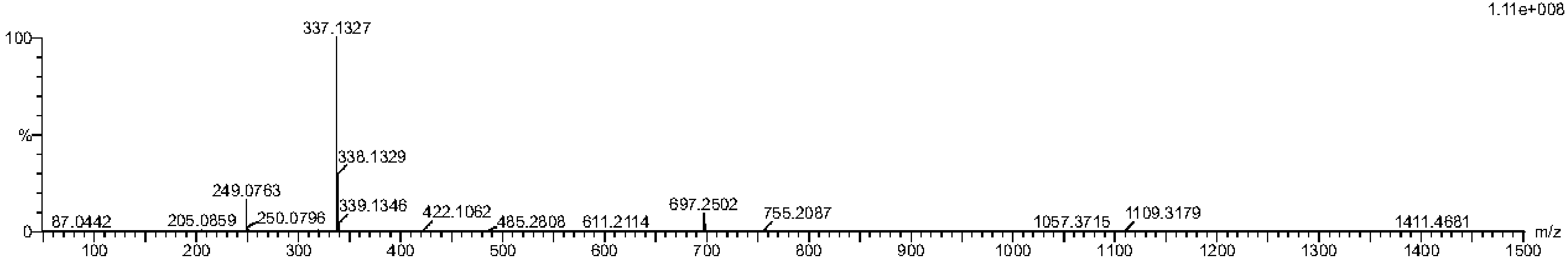

Minimum:	80.00				-1.5				
Maximum:	100.00		5.0	10.0	50.0				
Mass	RA	Calc. Mass	mDa	PPM	DBE	i-FIT	Norm	Conf(%)	Formula
337.1327	100.00	337.1287	4.0	11.9	7.5	2032.0	0.148	86.21	C17 H21 O7
		337.1346	-1.9	-5.6	-1.5	2033.8	1.981	13.79	C10 H25 O12

Figure S15. The HR-ESI-MS of compound **3**.

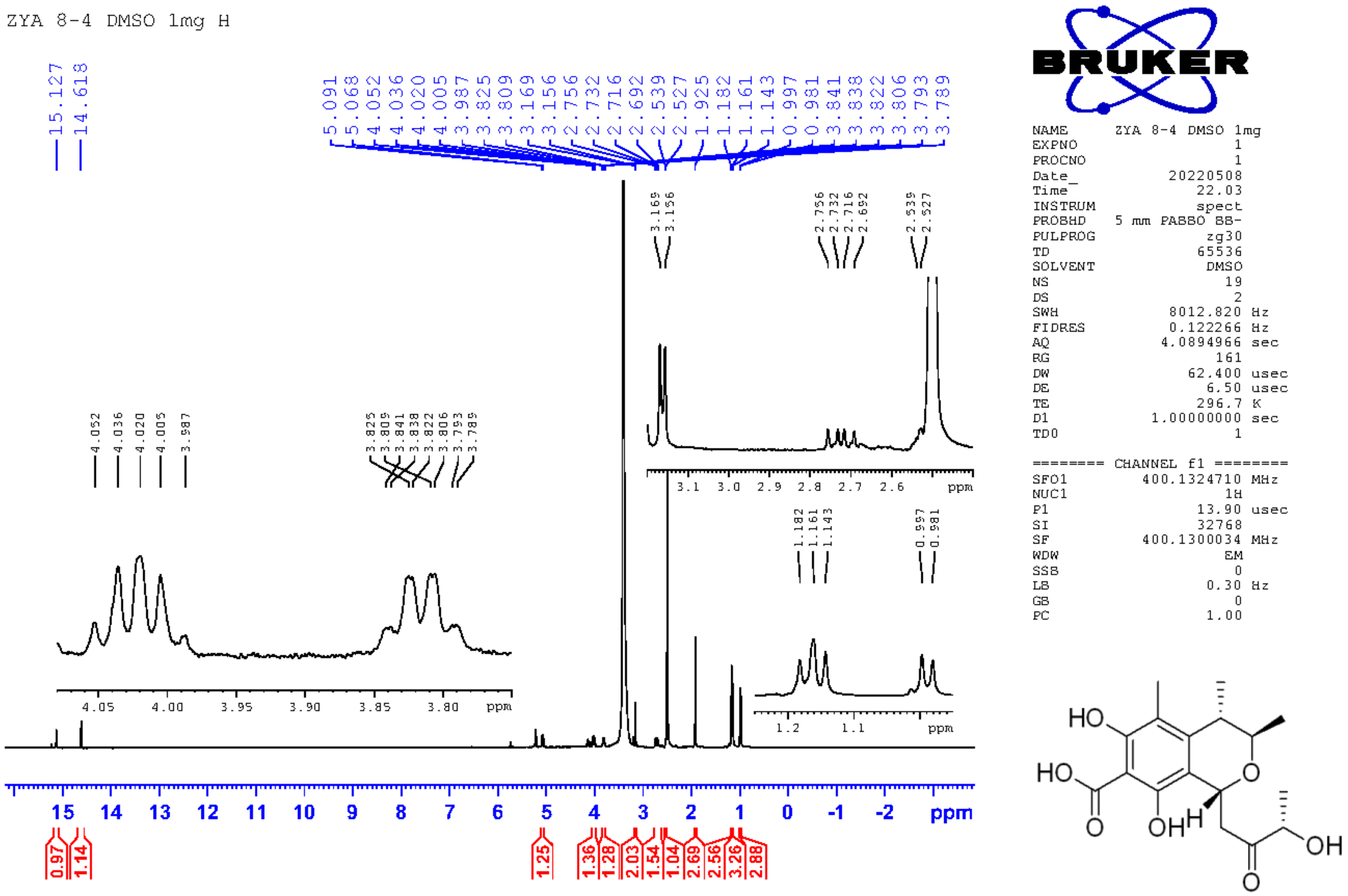

Figure S16. The ^{1}H NMR spectrum of compound **3** in DMSO-d_6.

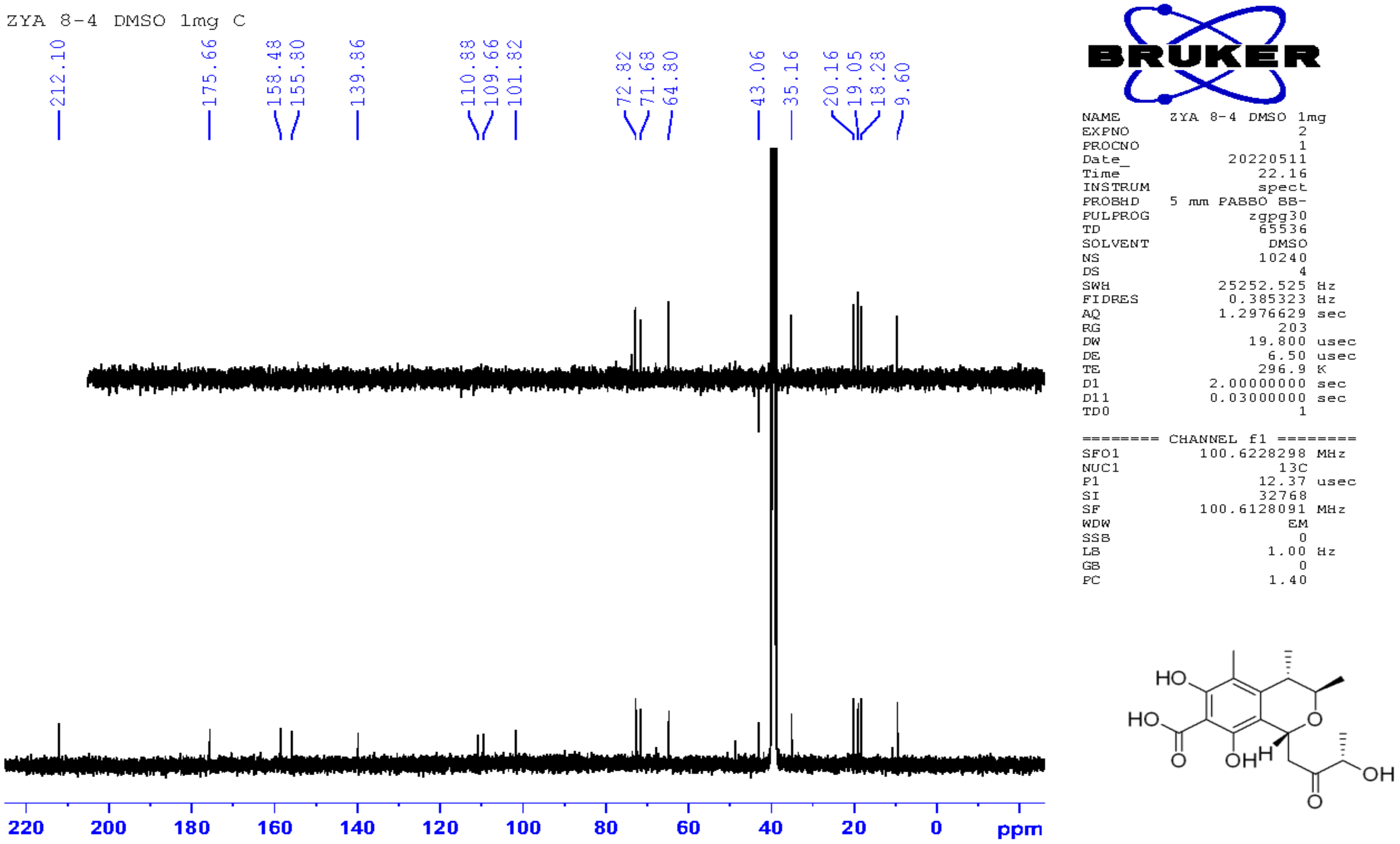

Figure S17. The ^{13}C NMR spectrum of compound **3** in DMSO-d_6.

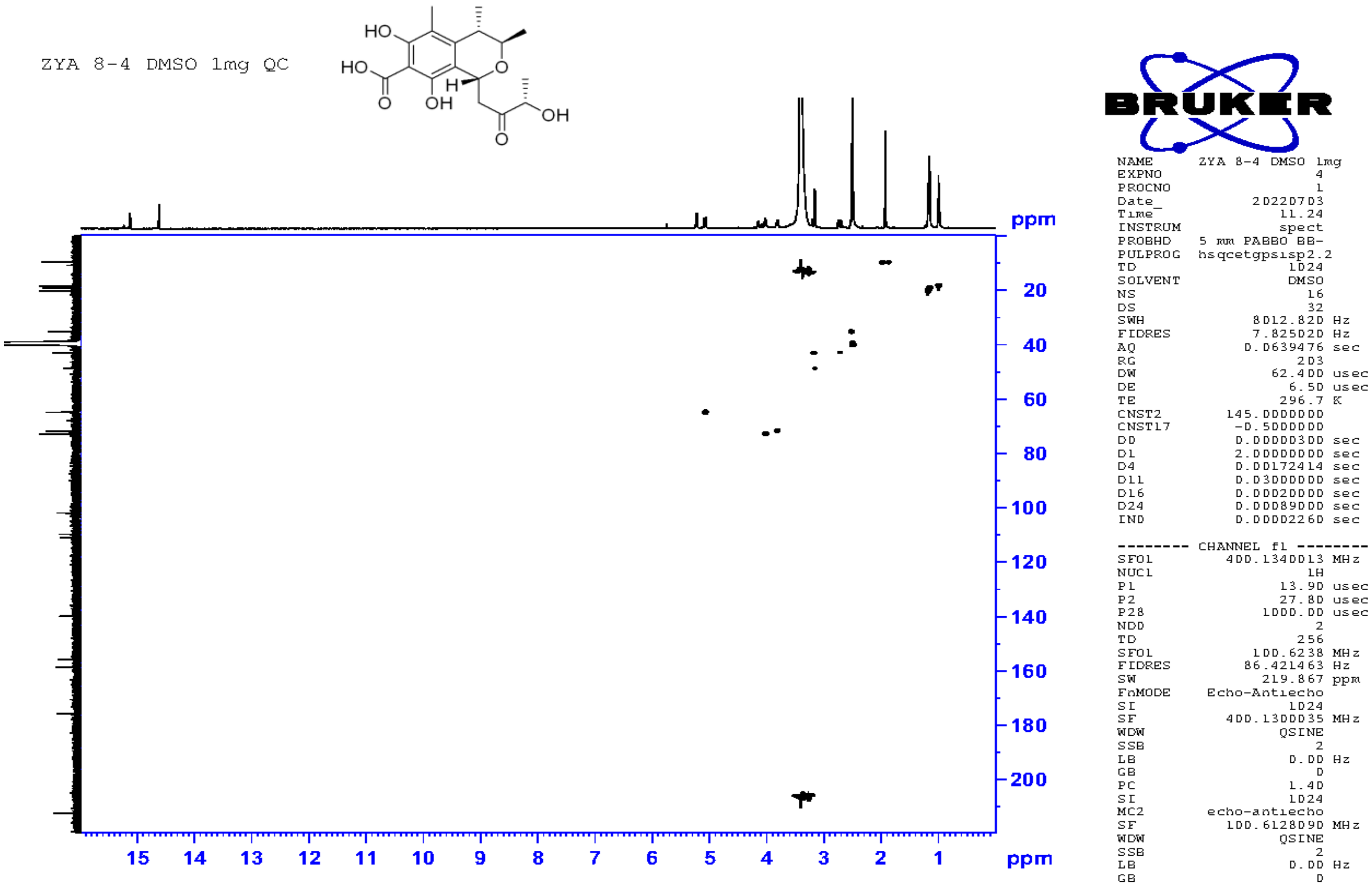

Figure S18. The HSQC spectrum of compound **3** in DMSO-d_6.

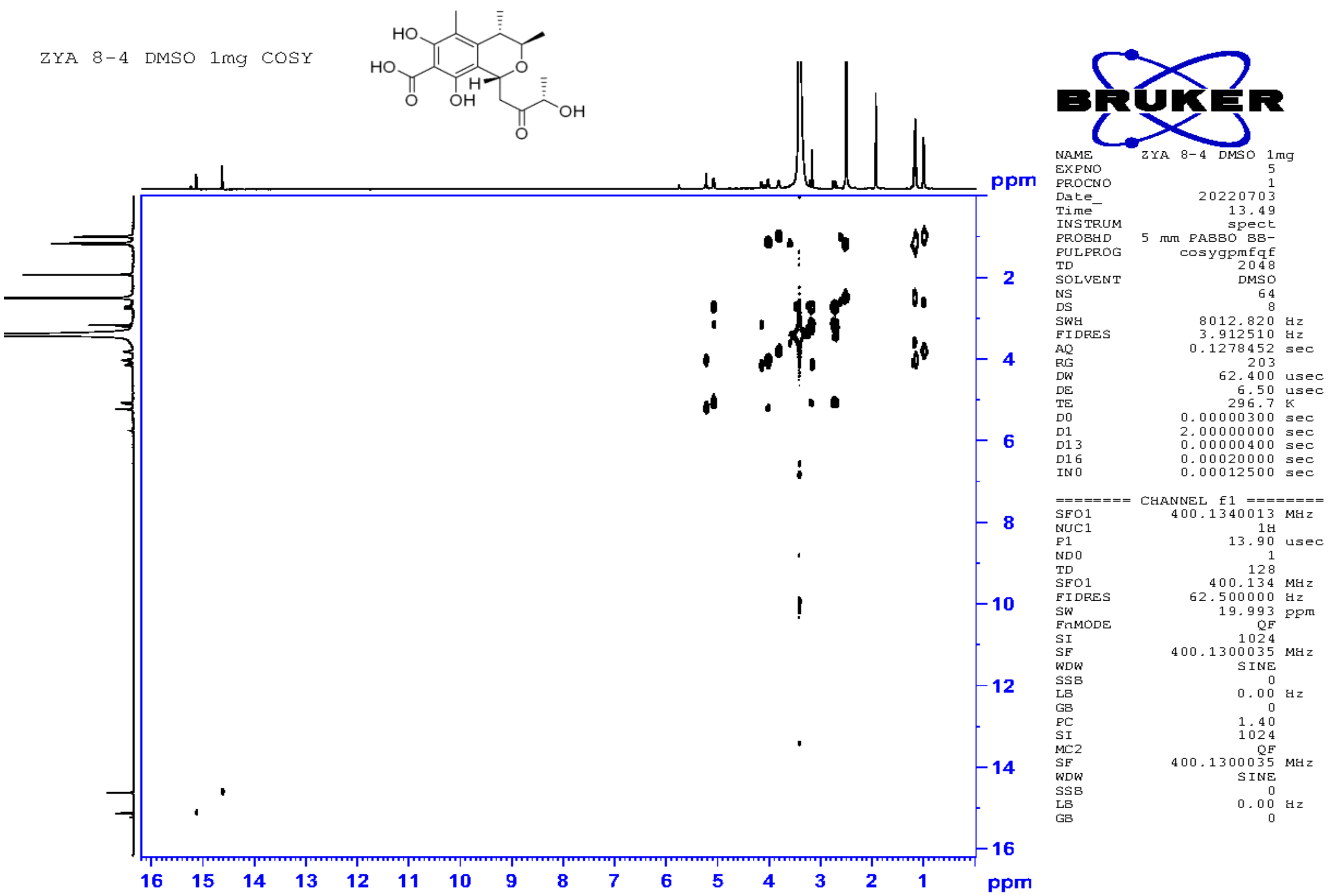

Figure S19. The ^{1}H-^{1}H COSY spectrum of compound **3** in DMSO-d_6.

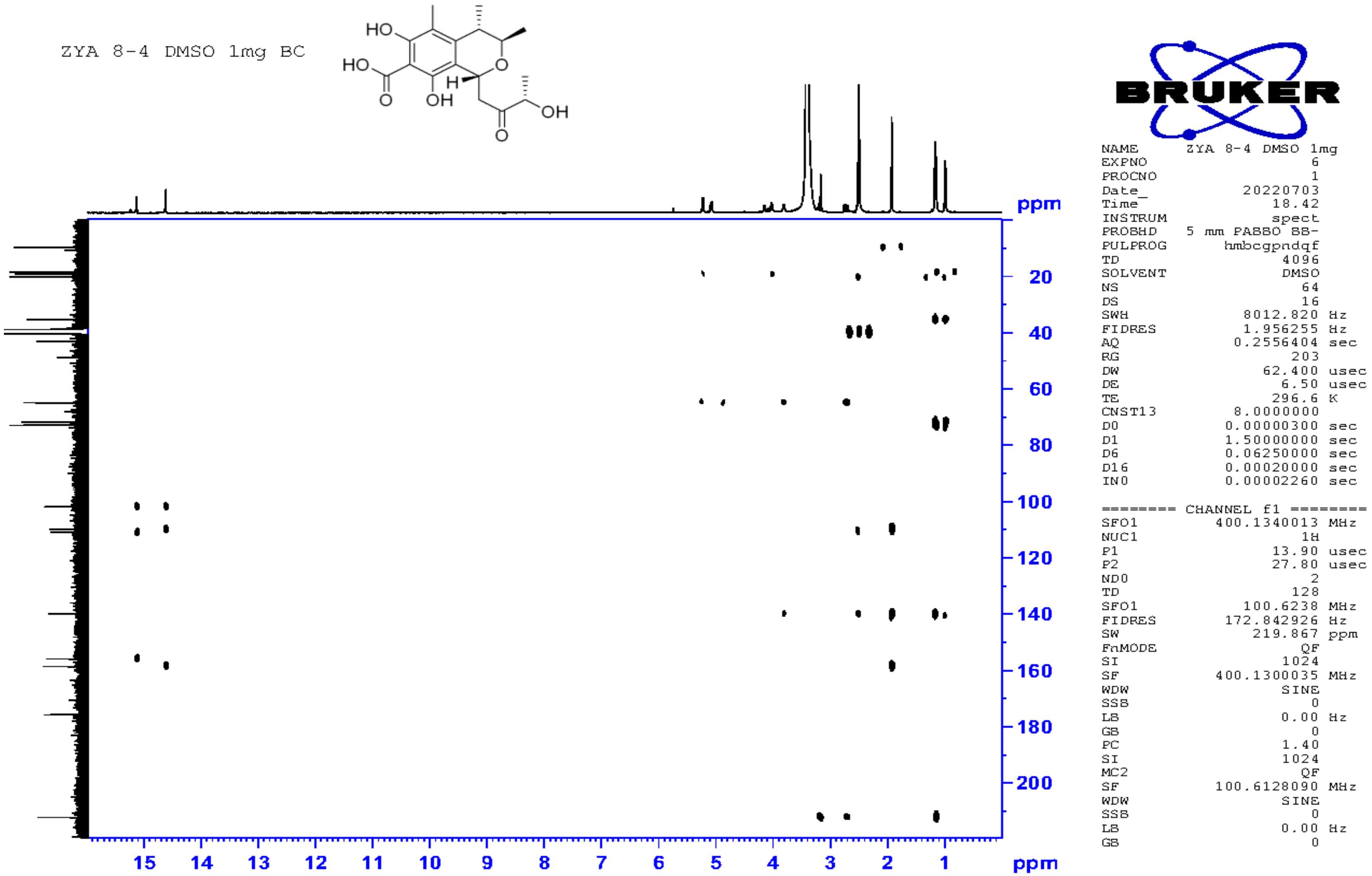

Figure S20. The HMBC spectrum of compound **3** in DMSO-d_6.

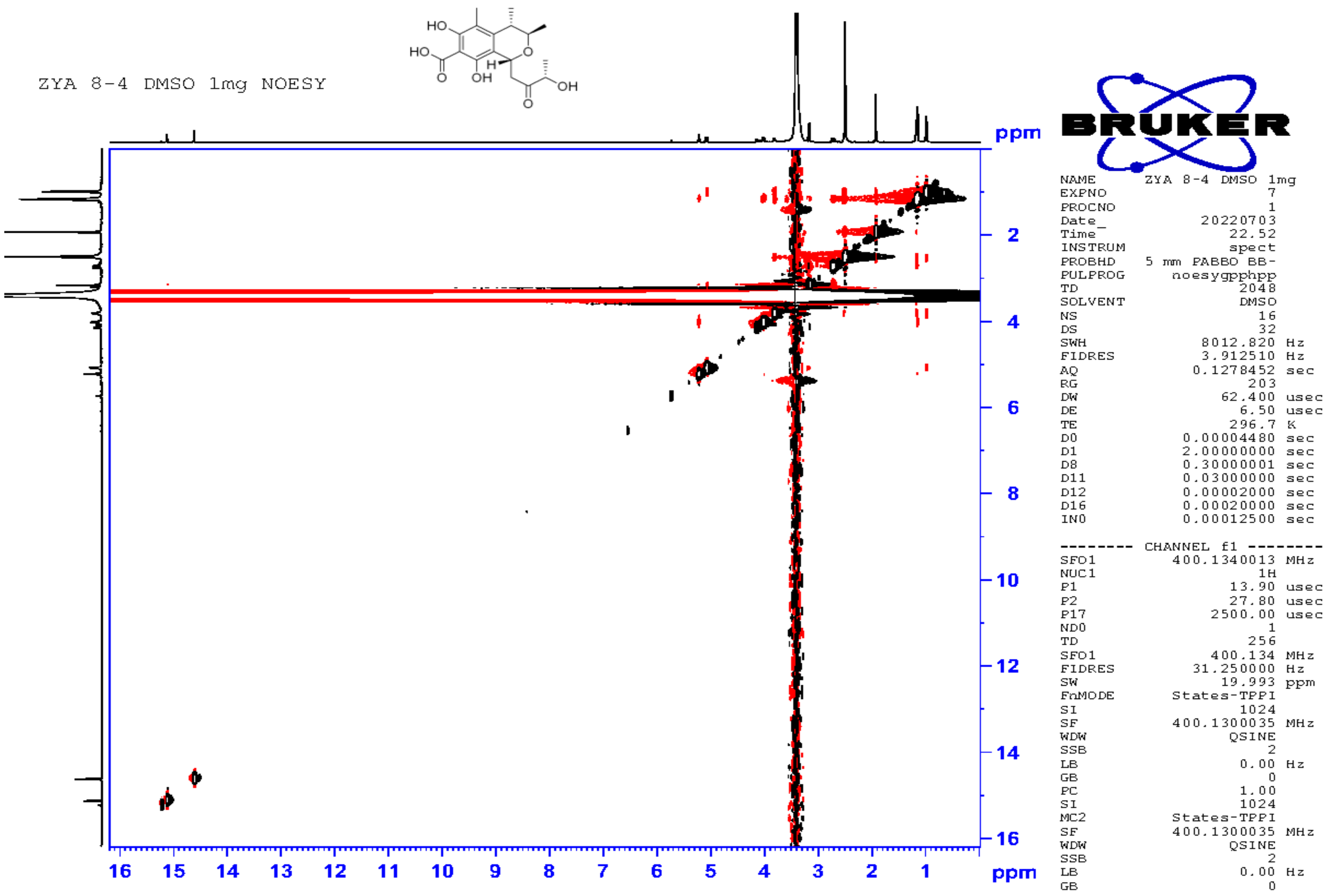

Figure S21. The NOESY spectrum of compound **3** in DMSO-d_6.
